# Effect of extracorporeal shock wave therapy on keratinocytes derived from human hypertrophic scars

**DOI:** 10.1038/s41598-021-96537-8

**Published:** 2021-08-27

**Authors:** Hui Song Cui, So Young Joo, Yoon Soo Cho, Ji Heon Park, Yu Mi Ro, June-Bum Kim, Cheong Hoon Seo

**Affiliations:** 1 Department of Rehabilitation Medicine, Burn Institute, Hangang Sacred Heart Hospital, College of Medicine, Hallym University, Seoul, 07247 Korea; 2 Department of Rehabilitation Medicine, Hangang Sacred Heart Hospital, College of Medicine, Hallym University, Seoul, 07247 Korea; 3 Department of Pediatrics, Hangang Sacred Heart Hospital, College of Medicine, Hallym University, Seoul, 07247 Korea

**Keywords:** Skin diseases, Pruritus, Skin manifestations, Cell biology, Energy

## Abstract

Hypertrophic scars represent a common complication in burn patients. In addition to cosmetic defects, they may cause serious sensory abnormalities such as pain and itching, severe dysfunction depending on the site, and emotional disorders such as anxiety and depression. The present study aimed to identify the molecular mechanisms underlying the use of extracorporeal shock wave therapy in keratinocytes. Keratinocytes derived from hypertrophic scar tissue were cultured and expression of proliferation markers (keratin 5 and 14), activation markers (keratin 6 and 17), differentiation markers (keratin 1, 10, and involucrin), apoptosis factors (Bax, Bcl2, and Caspase 14), and proliferation/differentiation regulators (p21 and p27) was investigated to compared with that of those in keratinocytes derived from normal skin tissue. Scar-derived keratinocytes were treated with extracorporeal shock waves under 1000 impulses at 0.1, 0.2, and 0.3 mJ/mm^2^. Shock waves altered the molecular pattern of proliferation, activation, differentiation, and apoptosis, as well as proliferation/ differentiation regulators, including Bax, Bcl2, ASK1, p21, p27, and Notch1. In summary, we show that extracorporeal shock wave therapy regulates the proliferation and differentiation of keratinocytes derived from hypertrophic scar to maintain normal epidermal integrity.

## Introduction

Hypertrophic scar hyperplasia is one of the most common complications after burn injury. Whereas the exact mechanism remains unknown, the phenomenon is characterized by the pathological proliferation of scar tissue caused by abnormal wound healing and scar maturation processes^1^. It is accompanied by excessive deposition of extracellular matrix, which not only increases scar thickness and decreases elasticity of the scar skin, but also causes continuous scar pain and itching in burn patients^1,2^. In addition, thicker scars in the joint area decrease the range of motion of the joint and become an important impediment to daily life movements and walking during the rehabilitation process^1^.

Various treatments, such as compression clothing, silicone gel, scar massage, laser treatment, intralesional injection, and surgical removal, are combined in clinical practice to treat hypertrophic scars in burn patients^3^. Triamcinolone is used for intralesional injection treatment, effectively reducing scar thickness and inhibiting proliferation^3^. However, as it affects also continuous progression of scar growth^3^, it is used only for localized scars. Therefore, a new, non-invasive, and effective treatment that can be applied continuously on a wide scar area is required.

Extracorporeal shock wave therapy (ESWT) is used to reduce inflammation, diminish pain, and regenerate soft tissues in musculoskeletal diseases^4^. A single treatment of ESWT upregulated with prolonged period time, 25–30 key pro-angiogenic gene expression that are decreased in both diabetic and wound healing models. ESWT inducing pro-angiogenic factors included for platelet–cell adhesion molecule‐1 (PECAM‐1), vascular endothelial growth factor (VEGF) and its receptor (VEGFR), endothelial nitric-oxide synthase (eNOS), and hypoxia-inducible factor 1α (HIF-1 α)^5^. ESWT not only induced endothelial cell proliferation, but also enhanced proliferation of other cell types^6–8^. Several previous studies both in vitro and vivo have shown an anti-inflammatory mechanism of ESWT, which treatment inhibited NF-κB activation and decreased oxygen radical production, leukocyte infiltration, chemokine expression, and pro-inflammatory cytokine expression^9–11^. In recent our study, shown ESWT down-regulated transforming growth factor beta 1 (TGF-β1) expression and reversed epithelial mesenchymal transition (EMT) in fibroblast derived from HTS^12^. The signal transduction associated with above events by ESWT is not clear. However, the activation of ERK1/2 or p38 MAPK may be responsible for the biological effects of ESWT^8,13,14^. Moreover, it has been reported to improve perfusion, ameliorate lymph circulation in patients with lymphedema, stimulate osteoblasts and union in fractures showing nonunion for more than 6 months, and promote regeneration of damaged peripheral nerves^15^. However, so far, the application of extracorporeal shock waves to treat hypertrophic scars resulting from burns has been limited to animal models^16^.

Although normal epithelial cells actively interact with interstitial fibroblasts, they come from most studies on the etiology of hypertrophic scars have revealed pathological proliferation of the dermis^1^. Although the normal epidermal keratinocytes actively interact with dermal fibroblasts^17^, little is known about whether keratinocytes seen in hypertrophic scars play a particular role in scar proliferation. A study using a tissue-engineered human skin model showed that pathological keratinocytes in the epidermis induced fibrotic endothelial matrix formation and inhibited extracellular matrix degrading factors, resulting in hypertrophic scar formation^18^. Therefore, examining the characteristics of epidermal tissue-derived keratinocytes, which many studies have focused on, will provide basic data for developing new treatments against thickening burn scars.

Previously, we reported the therapeutic effects of ESWT on scar pain and pruritus in burn patients with hypertrophic scar^19,20^, and showed that treatment changed the levels of hypertrophic scar-related molecules in fibroblasts derived from hypertrophic scars^12^. Examining the effect of extracorporeal shock waves on keratinocytes derived from hypertrophic scar tissue at the molecular level will provide important evidence about the effectiveness of this therapy for hypertrophic scar treatment.

## Methods

### Primary epidermal keratinocyte culture

All experimental protocols of this study were approved by Institutional Review Board of Hallym University Hangang Sacred Heart Hospital, and were carried out in compliance with the guidelines (Hallym University Hangang Sacred Heart Hospital Institutional Review Board 2014-062). Written informed consent was obtained from all subjects. After that, the procedure of primary cell culture was as published previously^21^. The hypertrophic scar tissue and normal skin tissue are paired from four patients that were obtained during surgical procedure, washed with 70% ethanol and cold phosphate-buffered saline containing antibiotics and antimycotics (Gibco, Life Technologies, Carlsbad, CA, USA). The tissue was cut into 2–4-mm-wide pieces, which were placed in 50-mL conical tubes containing 30 mL dispase II (0.5 mg/mL; Gibco) solution and agitated for 16 h at 4 °C. After digestion, the epidermis was obtained from the sample tissues by sterile forceps, and then digested with TrypLE™ Express Enzyme (Gibco) at 37 °C for 45 min. The solutions were added with KGM-Gold™ keratinocyte growth medium containing supplements (Lonza Bioscience, Basel, Switzerland) to the enzyme, and then filtered and centrifuged at 1500 rpm for 5 min. The pellet was suspended in the medium and cultivated at 37 °C in a 5% CO_2_ atmosphere. Human normal keratinocytes (HNKs) and human hypertrophic scar keratinocytes (HTSKs) were used for future experiments at passage 1 or 2.

### ESWT device and cell treatment

Keratinocytes were collected from cell culture dish by dissociated with Accutase^®^ cell detachment solution (Thermo Fisher Scientific, Waltham, MA, USA) and suspended in complete medium in a 4.5-cm-long conical tubes at 2.0 × 10^5^/mL. The cells treated a focused shock wave generated from an electromagnetic cylindrical coil source using Duolith SD-1^®^ device (StorzMedical, Tägerwilen, Switzerland). Cells were exposure to 1000 impulses/cm^2^ under an energy flux density (0.1, 0.2, and 0.3 mJ/mm^2^) and a frequency (4 Hz). After ESWT, that cells collected, centrifuged and further cultivated in complete medium in T75 culture plates. Cultivation for 24 h or 72 h, the cells collected, and used to analysis of mRNA and protein expression.

### Quantitative real-time PCR

Total RNA was extracted using a ReliaPrepTM RNA Miniprep system (Promega, Madison, WI, USA) according to the manufacturer’s instructions. RNA concentration was measured, cDNA was synthesized, and quantitative real-time PCR were performed as previously described^12,21^. The primer sequences are listed in Table 1. Target gene mRNA was normalized using the 2^−△△Ct^ method.

**Table 1:** Real-time PCR primer sequences.

Gene	Forward (5ʹ → 3ʹ)	Reverse (5ʹ → 3ʹ)
*KRT1*	TGGATGGTGCTTATATGAC	GACAACTCTGCTTGGTAG
*KRT5*	GTGGAAGACTTCAAGAACAA	TAGGCAGCATCTACATCC
*KRT6A*	TGAAGAAGGATGTGGATG	ATCATACAAGGCTCTCAG
*KRT10*	GATTCTCAACCTAACAACTG	GCTACCTCATTCTCATACT
*KRT14*	GCTGAGATCAAAGACTACA	AGAAGGACATTGGCATTG
*KRT17*	ATCCTGCTGGATGTGAAGACGC	TCCACAATGGTACGCACCTGAC
*IVL*	GGACTGCCTGAGCAAGAATGTG	TAA GCT GCT GCT CTG GGT TT
*NOTCH1*	AGCCTCAACGGGTACAAG	TTGACACAAGGGTTGGATTC
*CASP14*	CCTGTTGTCACCTTGCTAT	GTCCTTGCCTCTGTCTTAC
*BAX*	CCTTTTGCTTCAGGGTTTCA	CCATGTTACTGTCCAGTTCG
*BCL2*	TGCGGCCTCTGTTTGATTT	AGGCATGTTGACTTCACTTGT
*ACTB*	AGAGCTACGAGCTGCCTGAC	AGCACTGTGTTGGCGTACAG

### Western blotting analysis

HNKs or HTSKs were harvested and lysed in ice-cold RIPA buffer (Cell Signaling Technology, Danvers, MA, USA) pre-added a complete phosphatase inhibitor cocktail (Roche, Basel, Switzerland) and protease inhibitor cocktail (Sigma, St. Louis, MO, USA), and maintained for 1 h at 4 °C with constant agitation. The cell lysates were centrifuged for 15 min at 13,000×*g* and 4 °C, and the protein concentration was measured with a BCA kit (Thermo Fisher Scientific). Electrophoresis, primary and secondary antibody attachment, development (protein detection), and quantification were performed as previously described^12,21^. The primary antibodies are listed in Table 2. Target protein expression was normalized with β-actin, and expressed as ratio of HNK or un-treated controls (in ESWT treatment experiment).

**Table 2:** Western blot primary antibodies.

Target	Host	Dilution	Company (Cat. No.)
Keratin 1	Rabbit	1:1000	Abcam (ab93652)
Keratin 5	Rabbit	1:2000	Abcam (ab52635)
Keratin 6	Mouse	1:2000	Abcam (ab18586)
Keratin 14	Rabbit	1:2000	Abcam (ab181595)
Keratin 17	Rabbit	1:2000	Abcam (ab10975)
Involucrin	Mouse	1:500	Thermo Fisher Scientific (MA5-11803)
ASK 1	Rabbit	1:1000	Abcam (ab45178)
Notch 1	Rabbit	1:1000	Abcam (ab52627)
Caspase 14	Rabbit	1:1000	Abcam (ab174847)
Bax	Rabbit	1:1000	Abcam (ab199677)
Bcl2	Rabbit	1:1000	Abcam (ab196495)
P21	Rabbit	1:1000	Abcam (ab109199)
P27	Rabbit	1:1000	Abcam (ab32034)
β-actin	Rabbit	1:2000	Cell Signaling Technology (4967S)
β-actin	Mouse	1:1000	Santa Cruz Technology (sc-47778)

### Statistical analysis

All experimental results are presented with the mean ± standard deviation (SD). Comparisons between two groups were performed using the Mann–Whitney U test. Statistical analyses were performed using PASW statistics 24 (SPSS Inc., Chicago, IL, USA), and *P* < 0.05 was considered statistically significant.

## Results

### Characterization of HTSKs

**Figure 1:**
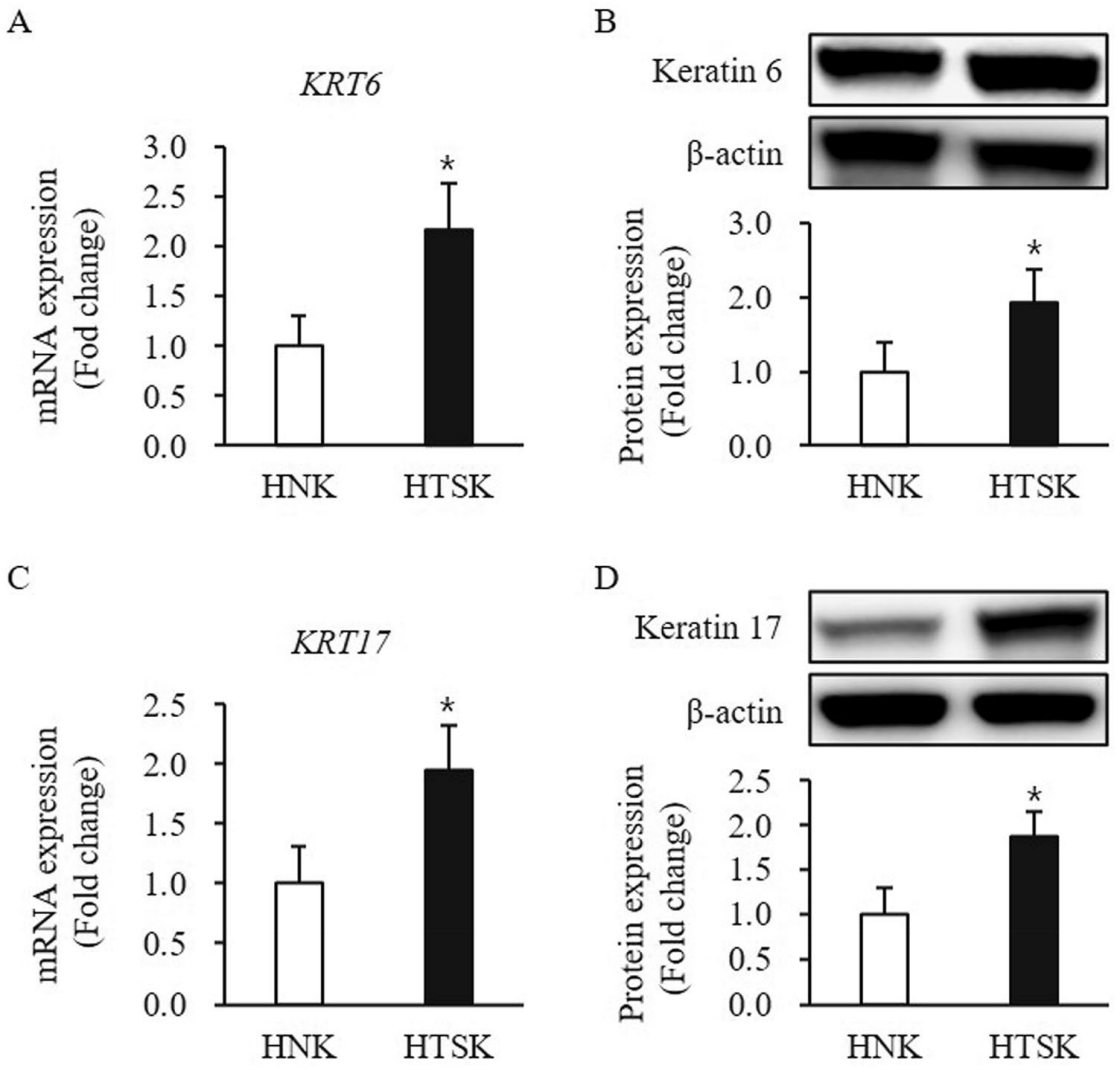
Expression of activation markers in HTSKs. Significantly increased mRNA levels of genes encoding keratin 6 (*KRT6*) **(A)** and keratin 17 (*KRT17*) **(C)** in HTSKs compared with those in HNKs. Significantly increased protein levels of keratin 6 **(B)** and keratin 16 **(D)** in HTSKs compared with those in HNKs. NHKs and HTSKs were primary cultured from normal skin tissues and HTS tissues, respectively. In the fold change, HNKs marked as value 1; HNK, human normal keratinocyte; HTSK, hypertrophic scar keratinocyte; *P < 0.05 for HTSK vs. the corresponding matched HNK. Data represent means ± SD; n = 4 (HNK) and n = 4 (HTSK).

A series of clear markers are expressed in the process of keratinocyte proliferation to differentiation. For example, classically considered that keratin 5 and 14 are proliferation marker, keratin 1, 10, and involucrin are differentiation marker, and keratin 6 and 16/17 are activation marker^22^. The expression of keratin 5 and 14 at mRNA and protein levels was no significant difference between HTSKs and HNKs (keratin 5—mRNA: 1.26 ± 0.37, protein: 1.206 ± 0.26-fold; keratin 14—mRNA: 1.16 ± 0.39, protein: 1.09 ± 0.38-fold, respectively; *P* > 0.05) (Figure S1A–D). The expression of keratin 6 and 17 at mRNA and protein was markedly higher in HTSKs than that of those in HNKs (keratin 6—mRNA: 2.16 ± 0.48, protein: 1.92 ± 0.46-fold increase; keratin 17—mRNA: 1.94 ± 0.38, protein: 1.83 ± 0.26—fold increase, respectively; *P* < 0.05) (Fig. 1).

**Figure 2:**
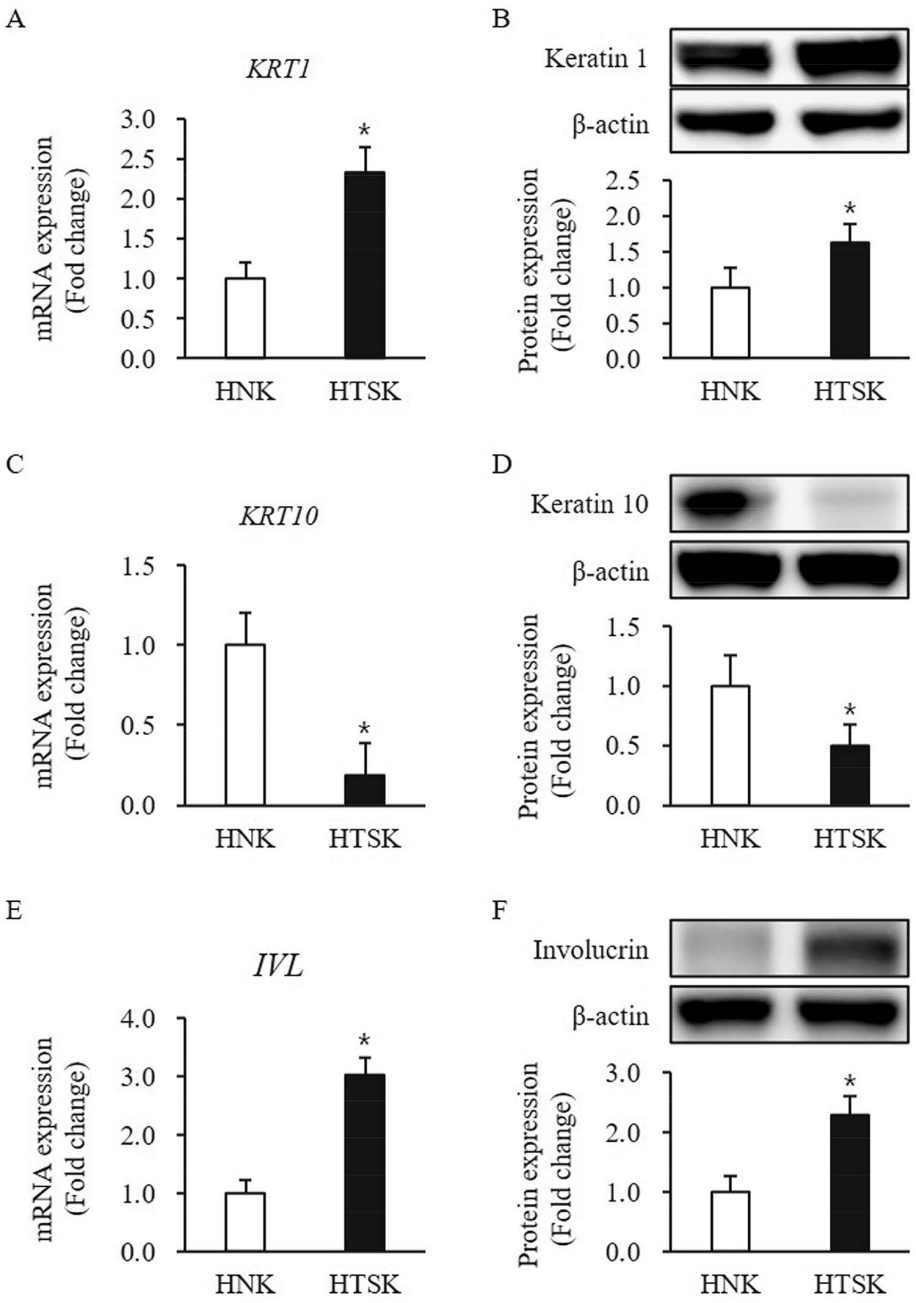
Expression of differentiation markers in HTSKs. Significantly increased mRNA levels of genes encoding keratin 1 (*KRT1*) **(A)**, decreased mRNA levels of genes encoding keratin 10 (*KRT10*) **(C)**, and increased mRNA levels genes encoding involucrin (*IVL*) **(E)** in HTSKs compared with those in HNKs. Significantly increased protein levels of keratin 1 **(B)** and decreased protein levels of keratin 10 **(D)** and increased protein levels of involucrin **(F)** in HTSKs compared with those in HNKs. NHKs and HTSKs were primary cultured from normal skin tissues and HTS tissues, respectively. In the fold change, HNKs marked as value 1; HNK, human normal keratinocyte; HTSK, hypertrophic scar keratinocyte; *P < 0.05 for HTSK vs. the corresponding matched HNK. Data represent means ± SD; n = 4 (HNK) and n = 4 (HTSK).

The mRNA and protein expression of keratin 1 was significantly higher in HTSKs than that of those in HNKs (mRNA: 2.33 ± 0.32, protein: 1.63 ± 0.26—fold increase, respectively; *P* < 0.05) (Fig. 2A,B). In contrast, the mRNA and protein expression of keratin 10 was significantly lower in HTSK than that of those in HNK (mRNA: 0.19 ± 0.19, protein: 0.50 ± 0.26—fold decrease, respectively; *P* < 0.05) (Fig. 2C,D). Moreover, involucrin expression at mRNA and protein levels was significantly higher in HTSKs than that of those in HNKs (mRNA: 3.03 ± 0.30, protein: 2.28 ± 0.31—fold increase, respectively; *P* < 0.05) (Fig. 2E,F).

**Figure 3:**
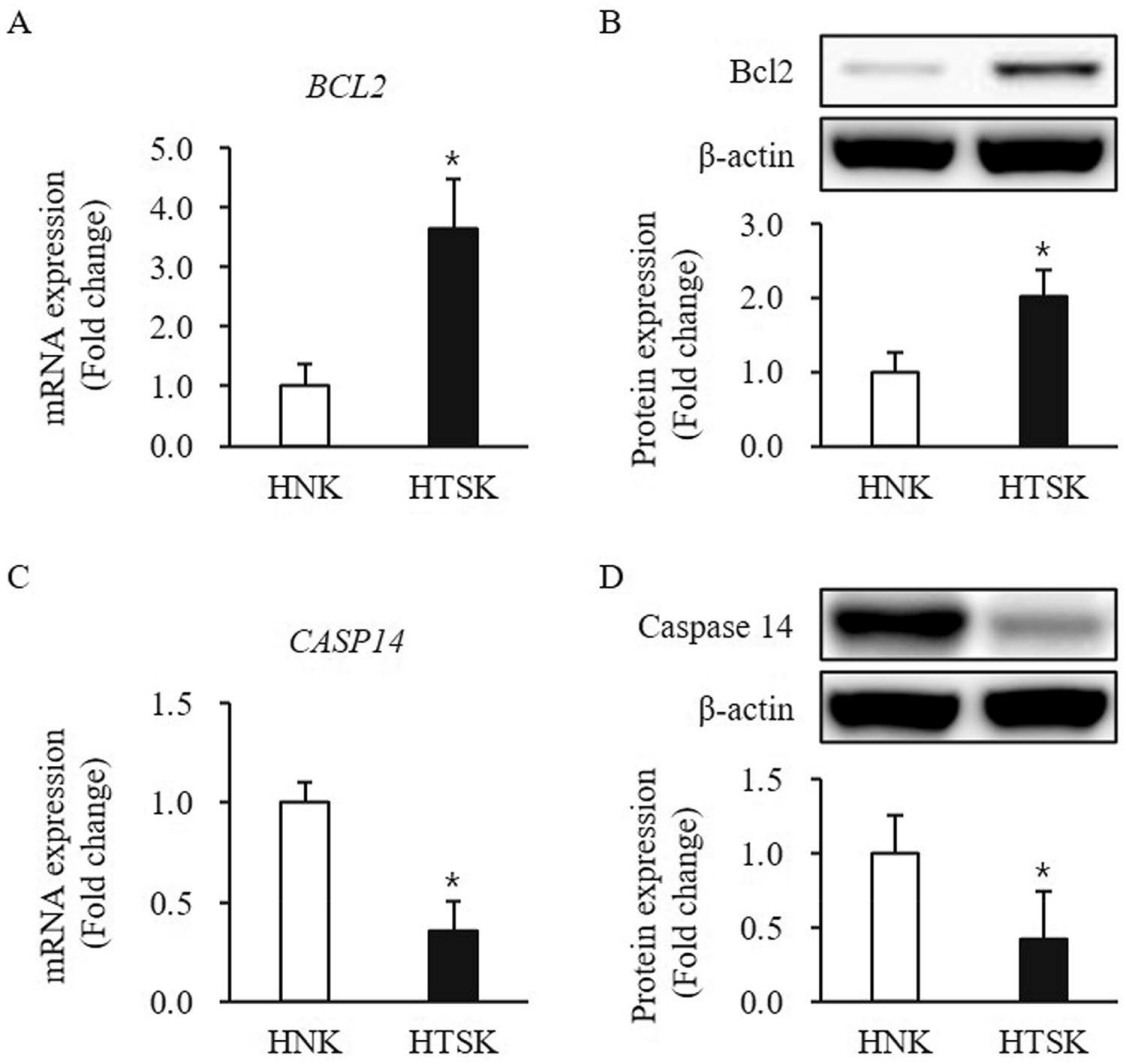
Expression of apoptosis-related factors in HTSKs. Significantly increased mRNA levels of genes encoding bcl2 (*BCL2*) **(A)** and decreased mRNA levels of genes encoding caspase 14 (*CASP14*) **(C)** in HTSKs compared with those in HNKs. Significantly increased protein levels of bcl2 **(B)** and decreased protein levels of caspase 14 **(D)** in HTSKs compared with those in HNKs. NHKs and HTSKs were primary cultured from normal skin tissues and HTS tissues, respectively. In the fold change, HNKs marked as value 1; HNK, human normal keratinocyte; HTSK, hypertrophic scar keratinocyte; *P < 0.05 for HTSK vs. the corresponding matched HNK. Data represent means ± SD; n = 4 (HNK) and n = 4 (HTSK).

The pro-apoptotic factor, Bax expression at mRNA and protein levels was slightly increased in HTSKs, but there is no significant difference between HTSKs and HNKs (mRNA: 1.23 ± 0.21, protein: 1.11 ± 0.14—fold increase, respectively; *P* > 0.05) (Figure S1E,F). In contrast, the anti-apoptotic factor, Bcl2 expression at mRNA and protein levels was significantly higher in HTSK than that of those in HNKs (mRNA: 3.65 ± 0.81, protein: 2.0 ± 0.36—fold increase, respectively; *P* < 0.05) (Fig. 3A,B). The mRNA and protein levels of caspase 14, which is a keratinocyte specific caspase was significantly lower in HTSKs than that of those in HNKs (mRNA: 0.355 ± 0.16, protein: 0.43 ± 0.22—fold decrease, respectively; *P* < 0.05) (Fig. 3C,D).

**Figure 4:**
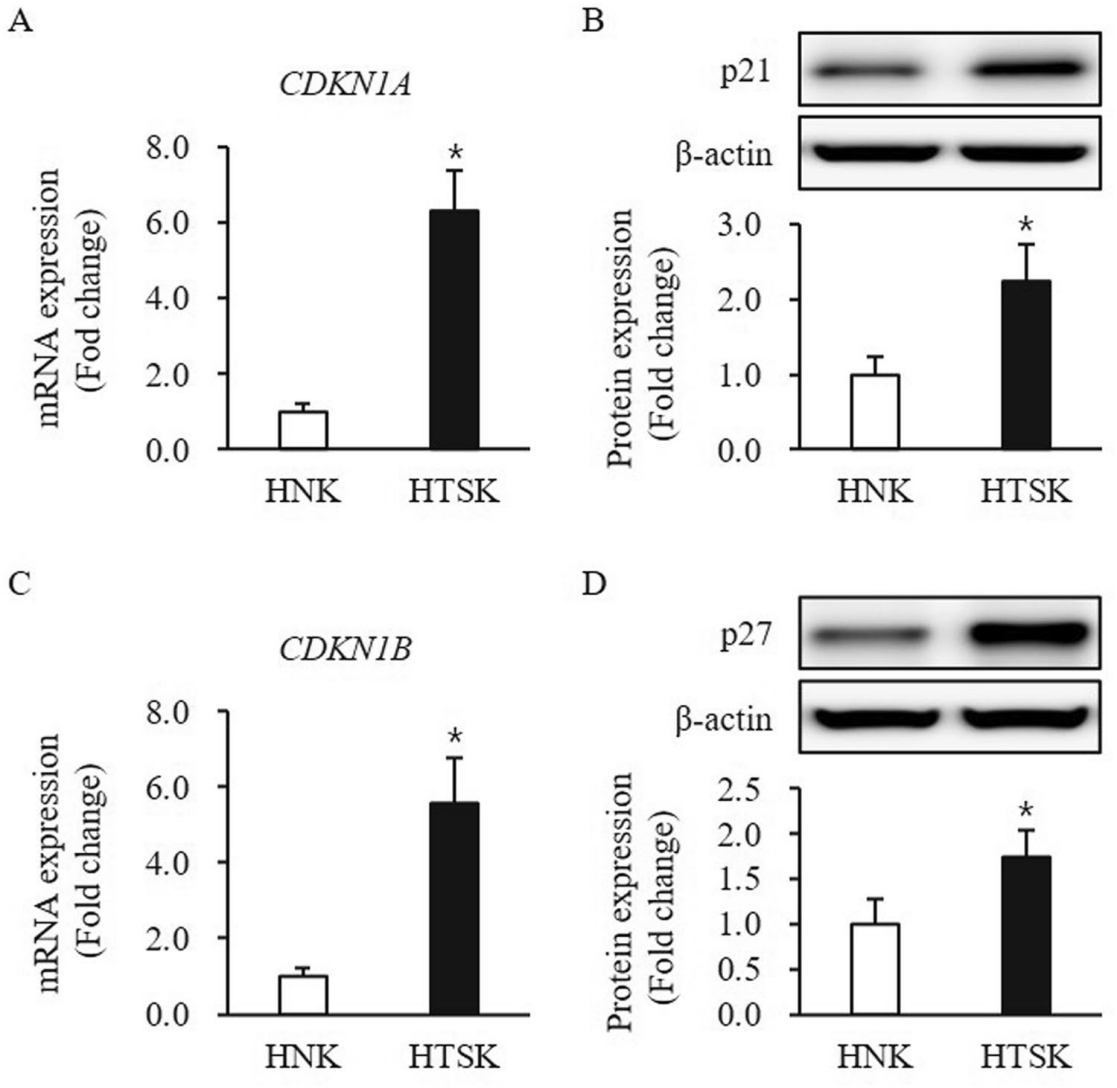
Expression of proliferation/differentiation regulators in HTSKs. Significantly decreased mRNA levels of genes encoding p21 (*CDKN1A*) **(A)** and p27 (*CDKN1B*) **(C)** in HTSKs compared with those in HNKs. Significantly decreased protein levels of p21 **(B)** and p27 **(D)** in HTSKs compared with those in HNKs. NHKs and HTSKs were primary cultured from normal skin tissues and HTS tissues, respectively. In the fold change, HNKs marked as value 1; HNK, human normal keratinocyte; HTSK, hypertrophic scar keratinocyte; *P < 0.05 for HTSK vs. the corresponding matched HNK. Data represent means ± SD; n = 4 (HNK) and n = 4 (HTSK).

The p21 and p27 are cell cycle regulator, to control proliferation to differentiation in keratinocyte, both were significantly higher at both mRNA and protein levels in HTSKs than that of those in HNKs (p21—mRNA: 6.32 ± 1.05, protein: 2.23 ± 0.49-fold increase; p27—mRNA: 5.55 ± 1.23, protein: 1.73 ± 0.30-fold increase, respectively; *P* < 0.05) (Fig. 4).

These results indicate that HTSKs are exhibiting an activated, over-differentiated, and augmented anti-apoptosis phenotype.

### Effect of ESWT on expression of proliferation and activation marker in HTSKs

**Figure 5:**
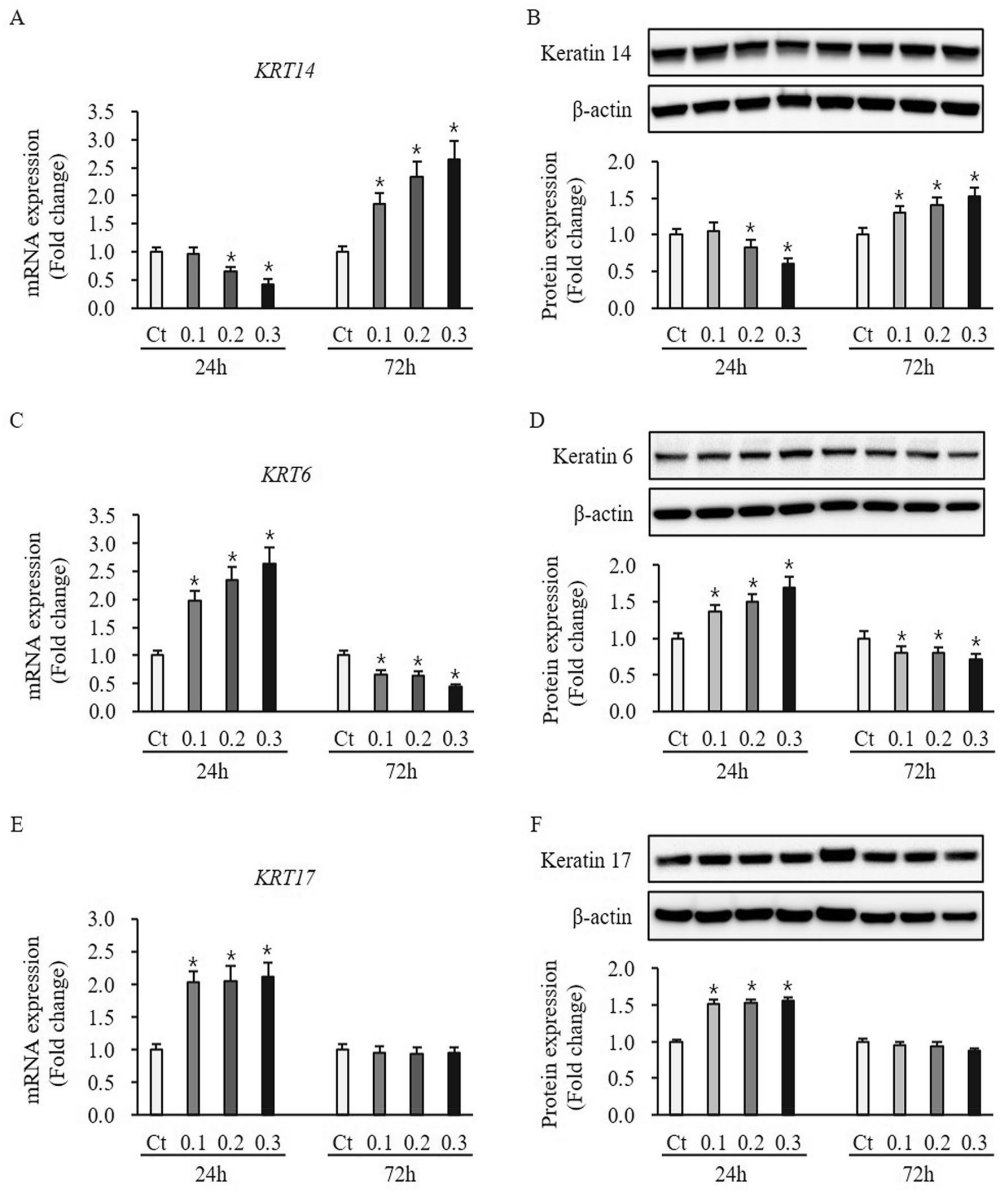
Expression of proliferation and activation marker in HTSKs after ESWT. Significantly decreased and increased mRNA **(A)** and protein levels **(B)** of keratin 14 (*KRT14*), a proliferation marker in HTSKs at 24 h or 72 h after ESWT under 0.2, and 0.3 mJ/mm^2^ of energy flux density, comparted with those in untreated control. Significantly increased and decreased mRNA **(C)** and protein levels **(D)** of keratin 6 (*KRT6*), an activation marker in HTSKs at 24 h or 72 h after ESWT under 0.1, 0.2, and 0.3 mJ/mm^2^ of energy flux density, comparted with those in untreated control. Significantly increased and unchanged mRNA **(E)** and protein levels **(F)** of keratin 17 (*KRT17*), anther activation marker in HTSKs at 24 h or 72 h after ESWT, comparted with those in untreated control. In the fold change, untreated control cells marked as value 1; Ct, untreated control cells; HTSK, hypertrophic scar keratinocyte; *P < 0.05 for ESWT-treated cells vs. the corresponding matched untreated control cells. Data represent means ± SD; n = 4.

ESWT had no effect on the expression of proliferation marker keratin 5 at mRNA and protein levels in HTSKs 24 h and 72 h after ESWT, compared to untreated control (mRNA: 0.1—0.97 ± 0.11, 0.2—0.95 ± 0.10, and 0.3—0.97 ± 0.10-fold at 24 h, 0.1—1.13 ± 0.09, 0.2—1.05 ± 0.12, and 0.3—1.17 ± 0.13-fold at 72 h, protein: 0.1—0.93 ± 0.09, 0.2—0.95 ± 0.10, and 0.3—0.99 ± 0.12-fold at 24 h, 0.1—0.99 ± 0.09, 0.2—1.01 ± 0.10, and 0.3—1.03 ± 0.11-fold at 72 h, respectively; *P* > 0.05) [Figure S2]. Another proliferation marker keratin 14 at mRNA and protein levels no difference between ESWT treatment and untreated control at 24 h after ESWT with 0.1 mJ/mm^2^ (mRNA: 0.1—0.96 ± 0.11, protein: 1.05 ± 0.12-fold at 24 h, *P* > 0.05) (Fig. 5A,B). However, keratin 14 at mRNA and protein levels in HTSKs 24 h and 72 h after ESWT with 0.2 and 0.3 mJ/mm^2^ significantly lower and higher than that of those in untreated control cells (mRNA: 0.2—0.65 ± 0.07 and 0.3—0.42 ± 0.10, protein: 0.2—0.80 ± 0.10 and 0.3—0.61 ± 0.08-fold decrease at 24 h, mRNA: 0.1—1.86 ± 0.19, 0.2—2.33 ± 0.29, and 0.3—2.64 ± 0.33, protein: 0.1—1.29 ± 0.10, 0.2—1.40 ± 0.11, and 0.3—1.53 ± 0.12-fold increase at 72 h, respectively; *P* < 0.05) (Fig. 5A,B). The activation marker keratin 6 at mRNA and protein levels in HTSKs 24 h and 72 h significantly higher and lower than that of those in untreated control cells (mRNA: 0.1—1.97 ± 0.18, 0.2—2.35 ± 0.22, and 0.3—1.62 ± 0.29-fold increase at 24 h, 0.1—0.66 ± 0.07, 0.2—0.63 ± 0.09, and 0.3—0.43 ± 0.05-fold decrease at 72 h, protein: 0.1—1.36 ± 0.10, 0.2—1.49 ± 0.11, and 0.3—1.69 ± 0.19-fold increase at 24 h, 0.1—0.80 ± 0.09, 0.2—0.78 ± 0.08, and 0.3—0.60 ± 0.08-fold decrease at 72 h, respectively; *P* < 0.05) (Fig. 5C,D).

Keratin 17 at mRNA and protein levels in HTSKs 24 h after all ESWT regimens significantly higher than that of those in untreated control cells (mRNA: 0.1—2.04 ± 0.15, 0.2—2.05 ± 0.21, and 0.3—2.12 ± 0.22-fold increase, protein: 0.1—1.51 ± 0.09, 0.2—1.53 ± 0.10, and 0.3—1.56 ± 0.10-fold increase at 24 h, respectively, *P* < 0.05) (Fig. 5E,F). However, they are no difference at 72 h after ESWT (mRNA: 0.1—0.96 ± 0.09, 0.2—0.93 ± 0.10, and 0.3—0.94 ± 0.09-fold, protein: 0.1—0.95 ± 0.11, 0.2—0.94 ± 0.12, and 0.3—0.88 ± 0.10-fold increase at 24 h, respectively, *P* > 0.05) (Fig. 5E,F). In addition, the protein expression of keratin 6, 14, and 17 in HNKs after ESWT was measured by western blotting. The proliferation marker keratin 14 expression was unaffected after all ESWT regimens (0.1—1.05 ± 0.08, 0.2—1.0 ± 0.09, and 0.3—1.02 ± 0.09-fold at 24 h, 0.1—0.99 ± 0.10, 0.2—1.02 ± 0.10, and 0.3—1.03 ± 0.10-fold at 72 h, respectively; *P* > 0.05) (Figure S3A). However, activation marker keratin 6 expression at protein levels in HNKs 24 h and 72 h after ESWT with 1000 impulses/cm^2^ at 0.1, 0.2. and 0.3 mJ/mm^2^ significantly higher and lower than that of those in untreated cells (0.1—1.33 ± 0.11, 0.2—1.54 ± 0.13, and 0.3—1.85 ± 0.19-fold increase at 24 h, 0.1—0.34 ± 0.04, 0.2—0.28 ± 0.03, and 0.3—0.27 ± 0.04-fold decrease at 72 h, respectively; *P* < 0.05) (Figure S3B). The keratin 17 is also an activation marker, which protein expression was unchanged after all ESWT regimens, compared to untreated control cells (0.1—1.04 ± 0.09, 0.2—1.05 ± 0.10, and 0.3—1.08 ± 0.10-fold at 24 h, 0.1—0.95 ± 0.10, 0.2—0.96 ± 0.09, and 0.3—0.94 ± 0.09-fold at 72 h, respectively; *P* > 0.05) (Figure S3C). These results suggest ESWT may regulate that HTSKs proliferation.

### Effect of ESWT on expression of differentiation marker in HTSKs

**Figure 6:**
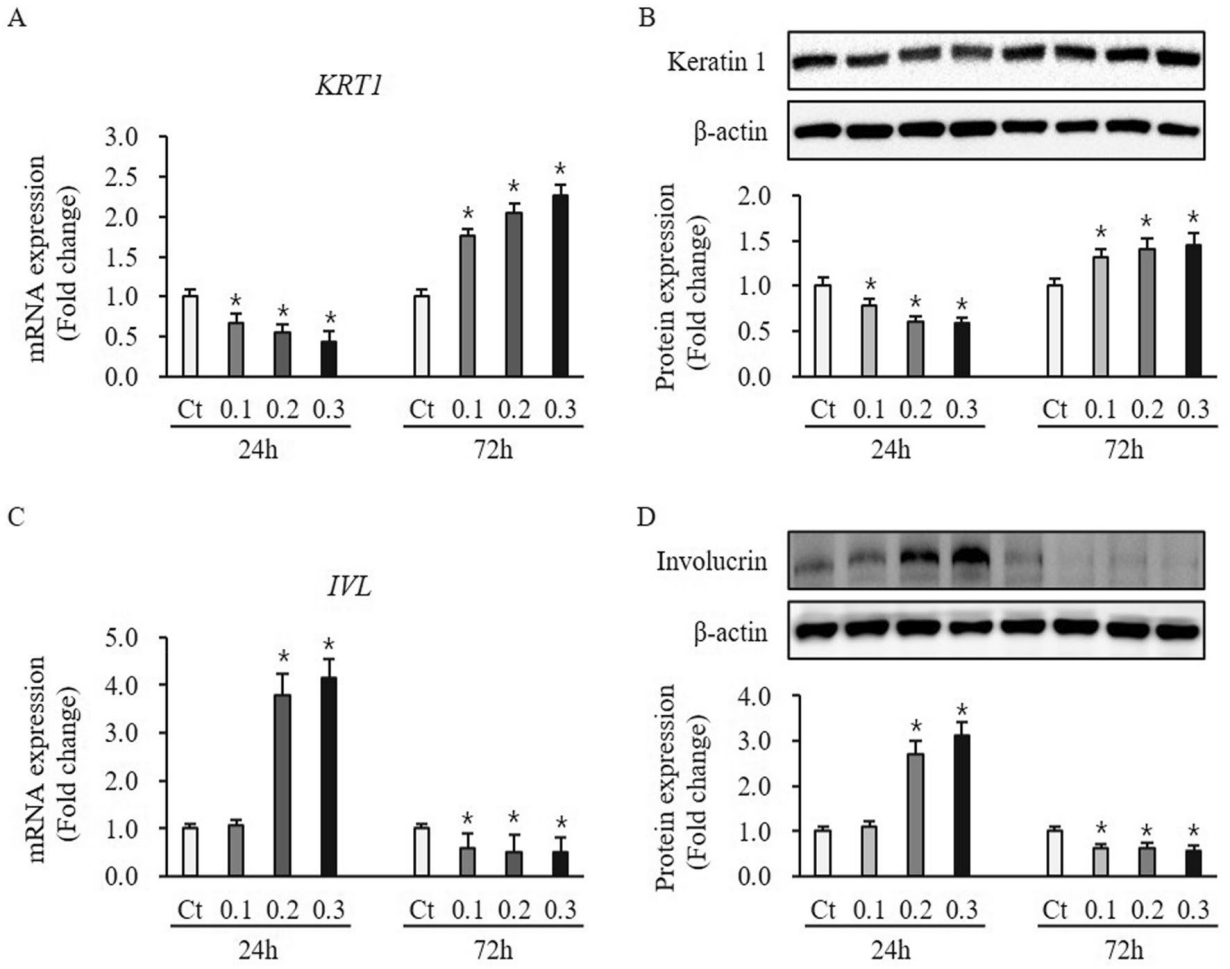
Expression of differentiation marker in HTSKs after ESWT. Significantly decreased and increased mRNA **(A)** and protein levels **(B)** of keratin 1 (*KRT1*) in HTSKs at 24 h or 72 h after ESWT under 0.1, 0.2, and 0.3 mJ/mm^2^ of energy flux density, comparted with those in untreated control. Significantly increased and decreased mRNA **(C)** and protein levels **(D)** of involucrin (*IVL*) in HTSKs at 24 h or 72 h after ESWT, comparted with those in untreated control. In the fold change, untreated control cells marked as value 1; Ct, untreated control cells; HTSK, hypertrophic scar keratinocyte; *P < 0.05 for ESWT-treated cells vs. the corresponding matched untreated control cells. Data represent means ± SD; n = 4.

The early differentiation marker keratin 1 expression at mRNA and protein levels in HTSKs 24 h and 72 h after ESWT significantly lower and higher than that of those in untreated control cells (mRNA: 0.1—0.67 ± 0.12, 0.2—0.55 ± 0.10, and 0.3—0.44 ± 0.12-fold decrease at 24 h, 0.1—1.76 ± 0.09, 0.2—2.05 ± 0.12, and 0.3—2.27 ± 0.13-fold increase at 72 h, protein: 0.1—0.78 ± 0.08, 0.2—1.61 ± 0.06, and 0.3—0.58 ± 0.07-fold decrease at 24 h, 0.1—1.32± 0.10, 0.2—1.41 ± 0.11, and 0.3—1.46 ± 0.12-fold increase at 72 h, respectively; *P* < 0.05) (Fig. 6A,B). The late differentiation marker involucrin expression at mRNA and protein levels in HTSKs 24 h after ESWT under 0.1 mJ/mm^2^ not significantly changed, when compared to that of those in untreated control cells (mRNA: 1.07 ± 0.12, protein: 1.09 ± 0.12, respectively; P > 0.05) (Fig. 6C,D). However, they at mRNA and protein levels in HTSKs 24 h after ESWT under 0.2 and 0.3 mJ/mm^2^ were significantly higher than that of those in untreated cells (mRNA: 0.2—3.77 ± 0.46, 0.3—4.15 ± 0.39, protein: 0.2—2.69 ± 0.30, 0.3—3.13 ± 0.28, respectively; *P* < 0.05) (Fig. 6C,D). Moreover, they at mRNA and protein levels 72 h after ESWT under all regimens were significantly lower than that of those in untreated control cells (mRNA: 0.1—0.59 ± 0.09, 0.2—0.50 ± 0.22, 0.3—0.49 ± 0.18, protein: 0.1—0.63 ± 0.09, 0.2—0.61 ± 0.12, 0.3—0.55 ± 0.14, respectively; *P* < 0.05) (Fig. 6C,D). In addition, protein expression of keratin 1 and involucrin in HNKs 24 h and 72 h after all ESWT regimens significantly higher and lower than that of those in untreated control cells, respectively (keratin 1: 0.1—1.35 ± 0.09, 0.2—1.49 ± 0.10, 0.3 – 1.43 ± 0.11-fold increase at 24 h, 0.1—0.31 ± 0.04, 0.2—0.39 ± 0.03, 0.3—0.37± 0.03-fold decrease at 72 h; keratin 17: 0.1—1.33 ± 0.11, 0.2—1.54 ± 0.13, 0.3—1.85 ± 0.18-fold increase at 24 h, 0.1—0.34 ± 0.04, 0.2—0.28 ± 0.03, 0.3—0.27± 0.04-fold decrease at 72 h, respectively; *P* < 0.05) (Figure S3 D and E).

### Effect of ESWT on expression of apoptosis-related factors in HTSKs

**Figure 7:**
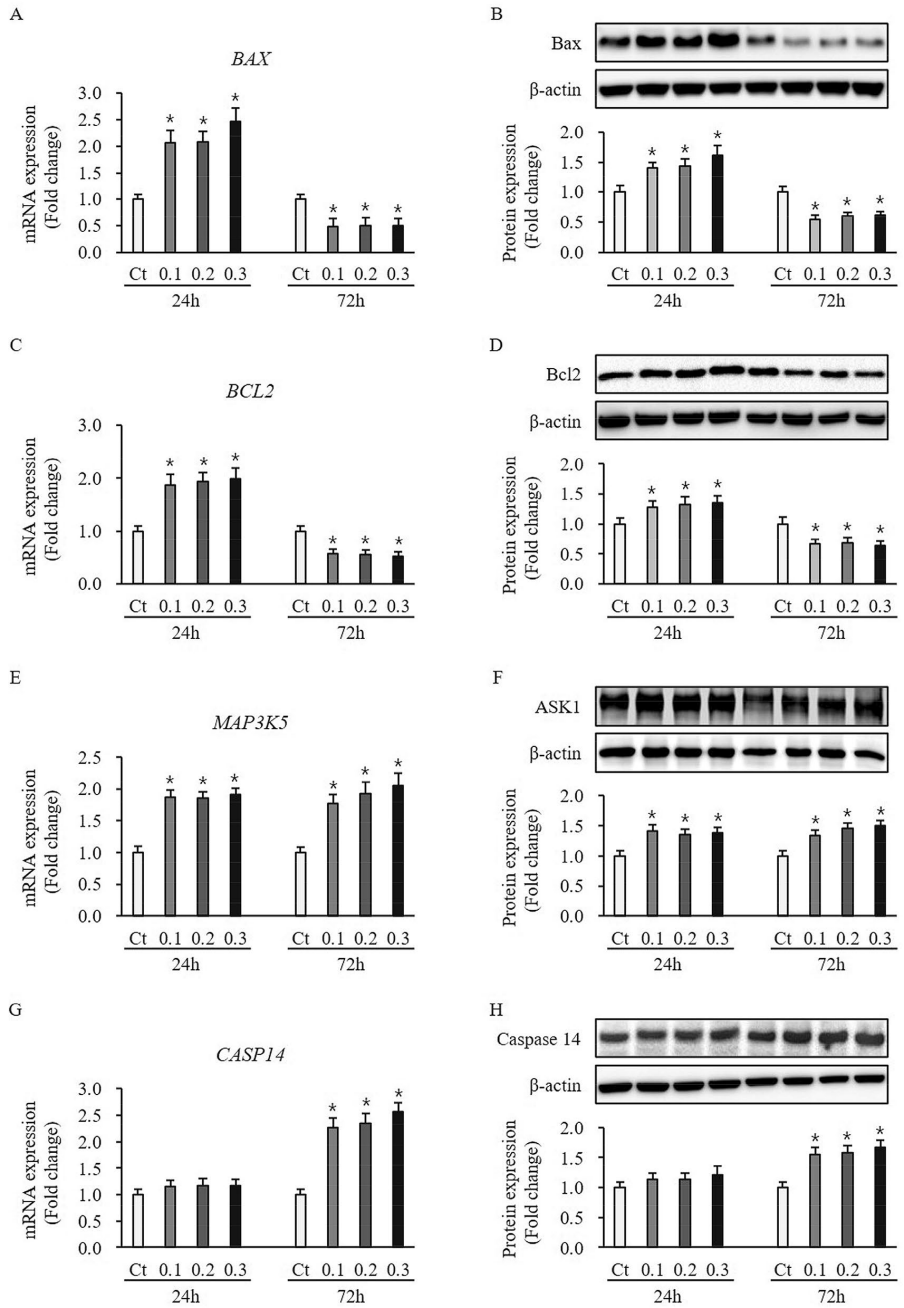
Expression of apoptosis-related factors in HTSKs. Significantly increased and decreased mRNA levels of bax (*BAX*) **(A)** and bcl2 (*BCL2*) **(C)** in HTSKs at 24 h or 72 h after ESWT under 0.1, 0.2, and 0.3 mJ/mm^2^ of energy flux density, comparted with those in untreated control. Significantly increased and decreased protein levels of bax **(B)** and bcl2 **(D)** in HTSKs at 24 h or 72 h after ESWT, comparted with those in untreated control. Significantly increased mRNA **(E)** and protein levels **(F)** of ASK1 (*MAP3K5*) in HTSKs at 24 h or 72 h after ESWT, comparted with those in untreated control. Unchanged and significantly increased mRNA **(G)** and protein levels **(H)** of caspase 14 (*CASP14*) in HTSKs at 24 h or 72 h after ESWT, comparted with those in untreated control. In the fold change, untreated control cells marked as value 1; Ct, untreated control cells; HTSK, hypertrophic scar keratinocyte; *P < 0.05 for ESWT-treated cells vs. the corresponding matched untreated control cells. Data represent means ± SD; n = 4.

The pro-apoptotic factor Bax expression at mRNA and protein levels in HTSKs 24 h and 72 h after ESWT significantly higher and lower than that of those in untreated control cells (mRNA: 0.1—2.06 ± 0.23, 0.2—2.08 ± 0.20, and 0.3—2.46 ± 0.26-fold increase at 24 h, 0.1—0.47 ± 0.16, 0.2—0.51 ± 0.15, and 0.3—0.50 ± 0.13-fold decrease at 72 h, protein: 0.1—1.40 ± 0.09, 0.2—1.43 ± 0.12, and 0.3—1.62 ± 0.15-fold increase at 24 h, 0.1—0.55 ± 0.07, 0.2—0.60 ± 0.05, and 0.3—0.61 ± 0.07-fold decrease at 72 h, respectively; *P* < 0.05) (Fig. 7A,B). Anti-apoptosis factor Bcl2 expression was in accordance with Bax expression at mRNA and protein levels (mRNA: 0.1—1.87 ± 0.20, 0.2—1.94 ± 0.17, and 0.3—1.99 ± 0.19-fold increase at 24 h, 0.1—0.56 ± 0.09, 0.2—0.55 ± 0.08, and 0.3—0.52 ± 0.10-fold decrease at 72 h, protein: 0.1—1.27 ± 0.10, 0.2—1.32 ± 0.13, and 0.3—1.35 ± 0.12-fold increase at 24 h, 0.1—0.67 ± 0.08, 0.2—0.69 ± 0.07, and 0.3—0.63 ± 0.09-fold decrease at 72 h, respectively; *P* < 0.05) (Fig. 7C,D). Apoptosis signal-regulating kinase 1 (ASK1) expression at mRNA and protein levels in HTSKs 24 h and 72 h after ESWT significantly higher than that of those in untreated control cells (mRNA: 0.1—1.87 ± 0.11, 0.2—1.85 ± 0.10, and 0.3—1.91 ± 0.10-fold increase at 24 h, 0.1—1.77 ± 0.14, 0.2—1.92 ± 0.19, and 0.3—2.06 ± 0.19-fold increase at 72 h, protein: 0.1—1.41 ± 0.11, 0.2—1.35 ± 0.09, and 0.3—1.38 ± 0.10-fold increase at 24 h, 0.1—1.34 ± 0.10, 0.2—1.46 ± 0.09, and 0.3—1.50 ± 0.10-fold increase at 72 h, respectively; *P* < 0.05) (Fig. 7E,F). Caspase 14 expression at mRNA and protein levels in HTSKs 24 h after ESWT was not significantly different from that of those in untreated control cells (mRNA: 0.1—1.15 ± 0.11, 0.2—1.17 ± 0.13, and 0.3—1.16 ± 0.12-fold, protein: 0.1—1.13 ± 0.10, 0.2—1.14 ± 0.11, and 0.3—1.21 ± 0.15-fold, respectively; *P* > 0.05) (Fig. 7G,H). However, they were significantly increased 72 h after treatment compared with untreated control cells (mRNA: 0.1—2.26 ± 0.19, 0.2—2.35 ± 0.18, and 0.3—2.57 ± 0.16-fold increase, protein: 0.1—1.55 ± 0.11, 0.2—1.58 ± 0.12, and 0.3—1.67 ± 0.13-fold increase, respectively; *P* < 0.05) (Fig. 7G,H). These results indicate ESWT may regulate HTSKs apoptosis.

### Effect of ESWT on expression of proliferation and differentiation regulators in HTSKs

**Figure 8:**
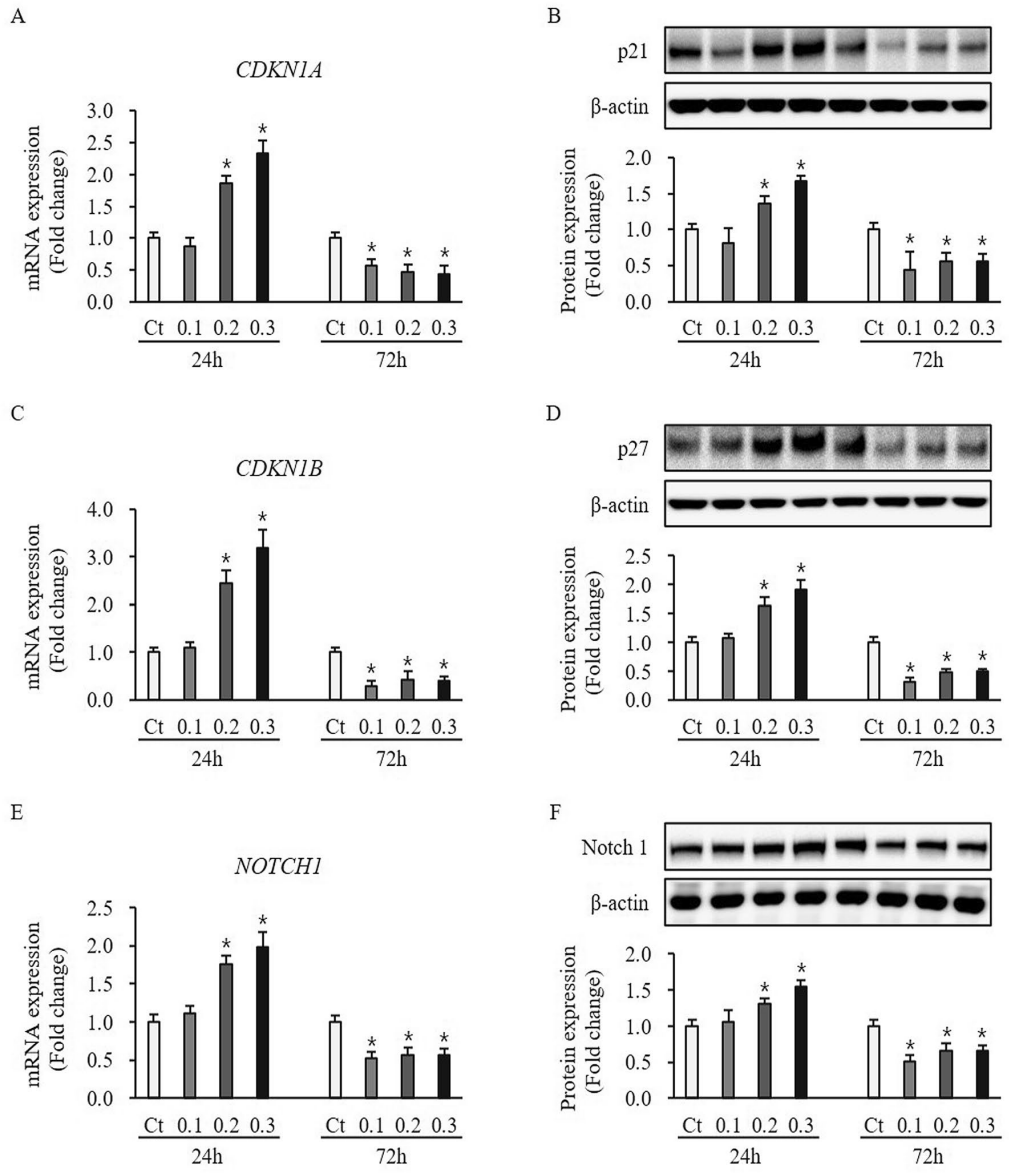
Expression of proliferation/differentiation regulators in HTSKs after ESWT. Significantly increased mRNA levels of genes encoding p21 (*CDKN1A*) **(A)**, p27 (*CDKN1B*) **(C)**, and Notch 1 (*NOTCH1*) **(E)** in HTSKs at 24 h after ESWT under 0.2 and 0.3 mJ/mm^2^ of energy flux density, compared with those in untreated control. Significantly decreased mRNA levels of genes encoding p21 (CDKN1A) **(A)**, p27 (CDKN1B) **(C)**, and Notch 1 (NOTCH1) **(E)** in HTSKs at 72 h after ESWT under 0.1, 0.2, and 0.3 mJ/mm^2^ of energy flux density, compared with those in untreated control. Significantly increased protein levels of p21 **(B)**, p27 **(D)**, and Notch 1 **(F)** in HTSKs at 24 h after ESWT under 0.2 and 0.3 mJ/mm^2^ of energy flux density, compared with those in untreated control. Significantly decreased protein levels of p21 **(B)**, p27 **(D)**, and Notch 1 **(F)** in HTSKs at 72 h after ESWT, compared with those in untreated control. In the fold change, untreated control cells marked as value 1; Ct, untreated control cells; HTSK, hypertrophic scar keratinocyte; *P < 0.05 for ESWT-treated cells vs. the corresponding matched untreated control cells. Data represent means ± SD; n = 4.

The p21, p27, and Notch1 expression at mRNA and protein levels in HTSKs 24 h after ESWT under 0.1 mJ/mm^2^ was not significantly changed, when compared with that of those in untreated cells (p21—mRNA: 0.1–0.87 ± 0.12, p27—mRNA: 0.1—1.10 ± 0.11, Notch 1—mRNA: 0.1—1.12 ± 0.09, p21—protein: 0.1—0.81 ± 0.21, p27—protein: 0.1—1.08 ± 0.07, Notch 1—protein: 0.1—1.06 ± 0.16, respectively; *P* > 0.05) (Fig. 8). However, they at mRNA and protein levels in HTSKs 24 h after ESWT under 0.2 and 0.3 mJ/mm^2^ were significantly higher than that of those in untreated cells (p21—mRNA: 0.2—1.86 ± 0.13, 0.3—2.34 ± 0.20, p27—mRNA: 0.2—2.44 ± 0.27, 0.3—3.18 ± 0.39; Notch 1—mRNA: 0.2—1.75 ± 0.11, 0.3—1.98 ± 0.20, p21—protein: 0.2—1.35 ± 0.11, 0.3—1.67 ± 0.08, p27—protein: 0.2—1.64 ± 0.14, 0.3—1.91 ± 0.16; Notch 1-protein 0.2—1.31 ± 0.08, 0.3—1.54 ± 0.09, respectively; *P* < 0.05) (Fig. 8). Moreover, they at mRNA and protein levels 72 h after ESWT under all regimens were significantly lower than that of those in untreated control cells (p21—mRNA: 0.1—0.57 ± 0.09, 0.2—0.46 ± 0.12, 0.3—0.44 ± 0.14, p27—mRNA: 0.1—0.29 ± 0.10, 0.2—0.43 ± 0.16, 0.3—0.40 ± 0.11; Notch 1—mRNA: 0.1—0.52 ± 0.09, 0.2—0.56 ± 0.11, 0.3—0.56 ± 0.11, p21—protein: 0.1—0.44 ± 0.25, 0.2—0.56 ± 0.12, 0.3—0.55 ± 0.10, p27—protein: 0.1—0.32 ± 0.07, 0.2—0.49 ± 0.06, 0.3—0.48 ± 0.05; Notch 1—protein: 0.1—0.51 ± 0.09, 0.2—0.66 ± 0.10, 0.3—0.65 ± 0.08, respectively; *P* < 0.05) (Fig. 8). These results indicate ESWT may regulate p21, p27, and Notch1 expression in HTSKs.

## Discussion

Despite the clinical studies indicate that ESWT benefit to patients with post-burn hypertrophic scar (HTS)^19,20,23,24^, However, the molecular mechanisms underlying observed beneficial effects after ESWT have remained unclear. In previous in vitro study in the fibroblasts derived from HTS, we demonstrated shock wave therapy had an antifibrotic effect^12^. Present study, we investigated the molecular changes of HTSKs after ESWT to provide an evidence for clinical application.

In the literature on the ESWT, no agreement on how to achieve maximum potential. There are several in vitro studies has been used with 100–300 pulses at 0.03–0.13 mJ/mm^2^ in mesenchymal cell line^14^, 1000 impulses at 0.14 mJ/ mm^2^ in human tenocytes^25^, and 0.06–0.50 mJ/mm^2^ in human osteoblasts^26^. Recent we applied ESWT with 4 Hz frequency, 0.05–0.30 mJ/mm^2^ energy flux density, and 1000 to 2000 impulses/cm^2^ per treatment according to patient's tolerance to pain. This treatment improved scar thickness, vascularity, and functionality, when treat to HTS of hand without obvious side effects^24^. Moreover, in our previous study, that ESWT with 1000 impulses/cm^2^ at 0.03, 0.1, and 0.3 mJ/mm^2^ not only altered the expression of fibrosis-related molecules in fibroblast derived from HTS, but did not affected the cells viability. Therefore, in this study, we selected the parameter with 1000 impulses/cm^2^ at 0.1, 0.2, and 0.3 mJ/mm^2^, applied to keratinocytes, and determined the molecular changes of cells.

At present, the fundamental epidermal pathology of HTS is still unclear. In previous studies, determined the expression of keratin 5 and 14, both are proliferation marker, and keratin 6, 16, and 17, they are activation marker by immunohistochemistry in HTS tissues that provided evidence that HTSKs were activated, but there was no difference in proliferation compared with site matched normal skin tissues^27^. Moreover, differentiation marker, filaggrin and involucrin were abnormally detected in HTS^27,28^. The increased expression of involucrin also found in other hyperproliferative disease, such as psoriasis^29^. The authors considered that abnormal expression of involucrin possibly affect stratum corneum formation^28^. In our results, the characteristic of HTSKs is similar to those of above previous study that is activated keratinocytes highly expressed keratin 6 and 17, and involucrin expression was also increased. Activated keratinocytes may play an important role in the development of HTS through the epidermal-mesenchymal interaction^17^. In HTSKs, the anti-apoptotic factor Bcl2 expression at mRNA and protein levels increased, while pro-apoptotic factor Bax expression was unchanged, compared to HNKs that suggest up-regulated anti-apoptosis in HTSKs. This results also reflect the low level of apoptosis is a cause of HTS formation^30^. The differentiation marker, keratin 1 and involucrin expression were increased in HTSKs, suggest differentiation of HTSKs was increased than HNKs. Moreover, the cell cycle regulator, p21 and p27 expression were also up-regulated in HTSKs. Study revelated the keratinocyte proliferation and differentiation were tightly regulated by the p21 and p27 expression. Moreover, over-expression of p21 and p27 induced keratinocyte differentiation and inhibited proliferation^31^. On the contrary, down-regulation of p21 and p27 expression resulted inhibition of differentiation with decrease of keratin-1 expression^32^. Therefore, increased expression of keratin 1 and involucrin may be related to up-regulated p21 and p27 expression in HTSKs.

We found the proliferation marker, keratin 14 expression was decreased at 24 h and increased at 72 h, while activation marker keratin 6 expression was increased at 24 h and decreased at 72 h. Moreover, early time differentiation marker keratin 1 expression was decreased at 24 h and increased at 72 h, and later differentiation marker, involucrin was increased at 24 h and decreased at 72 h. These results indicate that ESWT coordinately regulate HTSKs proliferation, activation, and differentiation, rather than blindly increase or decrease. Whereas, expression of keratin 6, and keratin 1 and involucrin in HNKs at 24 h after ESWT was increased and decreased at 72 h. These results suggest that ESWT exhibit different effects depending on physiological phenotypes. To maintain normal epidermal integrity, the proliferation-differentiation-death cycle must operate seamlessly. The several apoptosis-related factors physiologically involved in keratinocyte proliferation and differentiation^33^, including Bax, Bcl2, ASK1, and Caspase 14 etc. In normal epidermal development, Bcl-2 is highly expressed in basal proliferating keratinocytes and absent from suprabasal keratinocytes^34^. Bax expression has been reported to be stronger in suprabasal than in basal keratinocytes^35^. Over-expression of ASK1 in keratinocytes doses not induce apoptosis, but induce cell differentiation along with increasing the expression of involucrin^36^. Caspase-14 is uniquely expressed in skin tissues unlike other caspases type expressed ubiquitously^37^ and its accumulation is associated with keratinocyte differentiation and stratum corneum formation^38^. In our results, Bcl2 expression was correlated with Bax expression increased at 24 h decreased at 72 h after ESWT. Moreover, ASK1 expression was increased at 24 h and 72 h after ESWT. Caspase 14 expression was increased just at 72 h after treatment. These results are further supported the effect of shock wave therapy regulate HTSKs proliferation and differentiation. To sum up the above, that over- or down-expression of apoptosis-related factors reflects ESWT treatment induces the proliferation or differentiation phenotype keratinocyte.

Overall, the shock wave therapy altered the expression of proliferation and differentiation—related molecular in HTSKs. The increased or decreased expression of molecular in HTSKs 24 h or 72 h after treatment was close to that of those in the normal cell; for instance, keratin 1 expression at 24 h, involucrin, bcl2, p21, and p27 expression at 72 h vs. that of those in HNKs. The consequence its dynamic regulation may be partially involved in pathological phenotype restore to normal cell physiological state contribute to maintain normal epidermal homeostasis.

Notch1, p21, and p27 are also involved in regulating the proliferation and differentiation of keratinocytes^39–42^. Specifically, activation of Notch1 upregulation of p21 to induce differentiation associated cell arrest, whereas p27 activation promotes the differentiation of keratinocytes^40–42^. In our results, expression of Notch1, p21, and p27 was increased at 24 h and decreased at 72 h after ESWT. Their expression pattern was consistent with that expression of keratin 14 and involucrin. Therefore, the effect of ESWT on the proliferation and differentiation of HTSKs possibly through regulate expression of Notch1, p21, and p27.

In summary, present study demonstrated that extracorporeal shock wave dynamically regulates proliferation, activation, and differentiation of keratinocytes originated from hypertrophic scar tissue (Table 3). This evidence partially supports observed in clinical beneficial effects of shock wave therapy for post-burn hypertrophic scar. Future study will be carried out in a three-dimensional culture model to fully explore the mechanism of action and to provide more physiologically relevant information.

**Table 3:** Impact of the effect of ESWT on human hypertrophic scar keratinocytes.

Marker type	Protein and gene of interest	HTSKs compared to HNKs	After treatment 24 h	After treatment 72 h
Proliferation	Keratin 5 (*KRT5*)	n.c	n.c	n.c
Proliferation	Keratin 14 (*KRT14*)	n.c	−	+
Activation	Keratin 16 (*KRT6*)	+	+	−
Activation	Keratin 17(*KRT17*)	+	+	n.c
Differentiation	Keratin 1 (*KRT1*)	+	−	+
Differentiation	Keratin 10 (*KRT10*)	−	n.c	n.c
Differentiation	Involucrin (*IVL*)	+	+	−
Pro-apoptotic	Bax (*BAX*)	– –	+	^−^
Anti-apoptotic	Bcl2 (*BCL2*)	+ +	+	−
Pro-apoptotic	Caspase 14 (*CASP14*)	– –	n.c	+
Cell cycle regulator	p21 (*CDKN1A*)	+ +	+	−
Cell cycle regulator	p27 (*CDKN1B*)	+ +	+	−

+ indicates fold change from control < 2.0.

+  + indicates fold change from control > 2.0.

− indicates fold change from control 0.0 to −0.5.

– – indicates fold change > −0.5.

*n.c* not changed.

## Supplementary Information

Supplementary Figures.
