# Supplementary material for: Effect of extracorporeal shock wave therapy on keratinocytes derived from human hypertrophic scars
Source: Sci Rep. 2021 Aug 27;11:17296. doi: 10.1038/s41598-021-96537-8 (PMC8397706; doi:10.1038/s41598-021-96537-8)

**Effect of extracorporeal shock wave therapy on keratinocytes derived from human hypertrophic scars**

Hui Song Cui ^1†^, So Young Joo ^2†^, Yoon Soo Cho ^2^, Ji Heon Park ^1^, Yu Mi Ro^1^, June-Bum Kim ^3^*, and Cheong Hoon Seo ^2^*

Supplementary Information


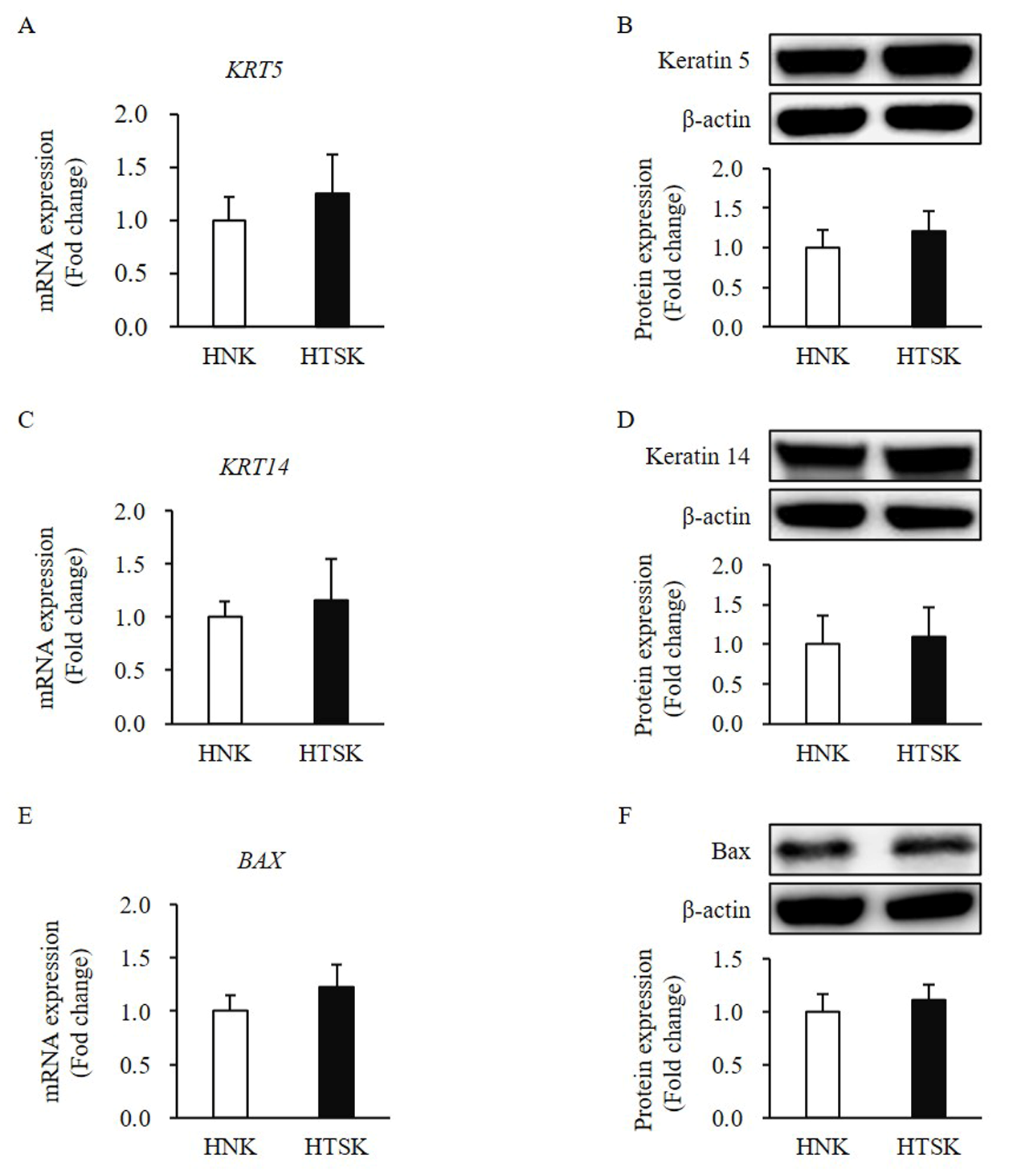


Figure S1. Unchanged mRNA levels of keratin 5 and 14, and Bax in HTSKs compared with those in HNKs (A, C, and E). Unchanged protein levels of keratin 5 and 14, and Bax in HTSKs compared with those in HNKs (B, D, and F). In the fold change, HNKs marked as value 1; HNK, human normal keratinocyte; HTSK, hypertrophic scar keratinocyte; Data represent means ± SD; n = 4 (HNK) and n = 4 (HTSK).


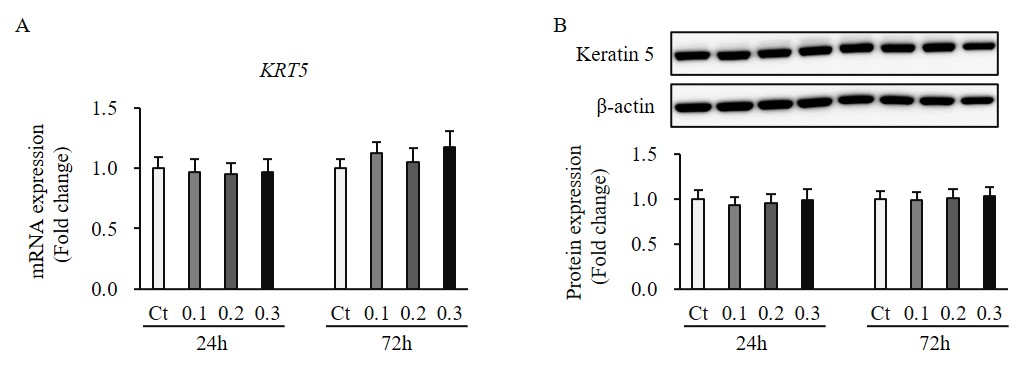


Figure S2. Unchanged mRNA (A) and protein (B) levels of keratin 5 (*KRT5*) in HTSKs at 24h or 72h after ESWT under 0.1, 0.2, and 0.3 mJ/mm^2^ of energy flux density, comparted with those in untreated control. In the fold change, untreated control cells marked as value 1; Ct, untreated control cells; HTSK, hypertrophic scar keratinocyte; Data represent means ± SD; n = 4.


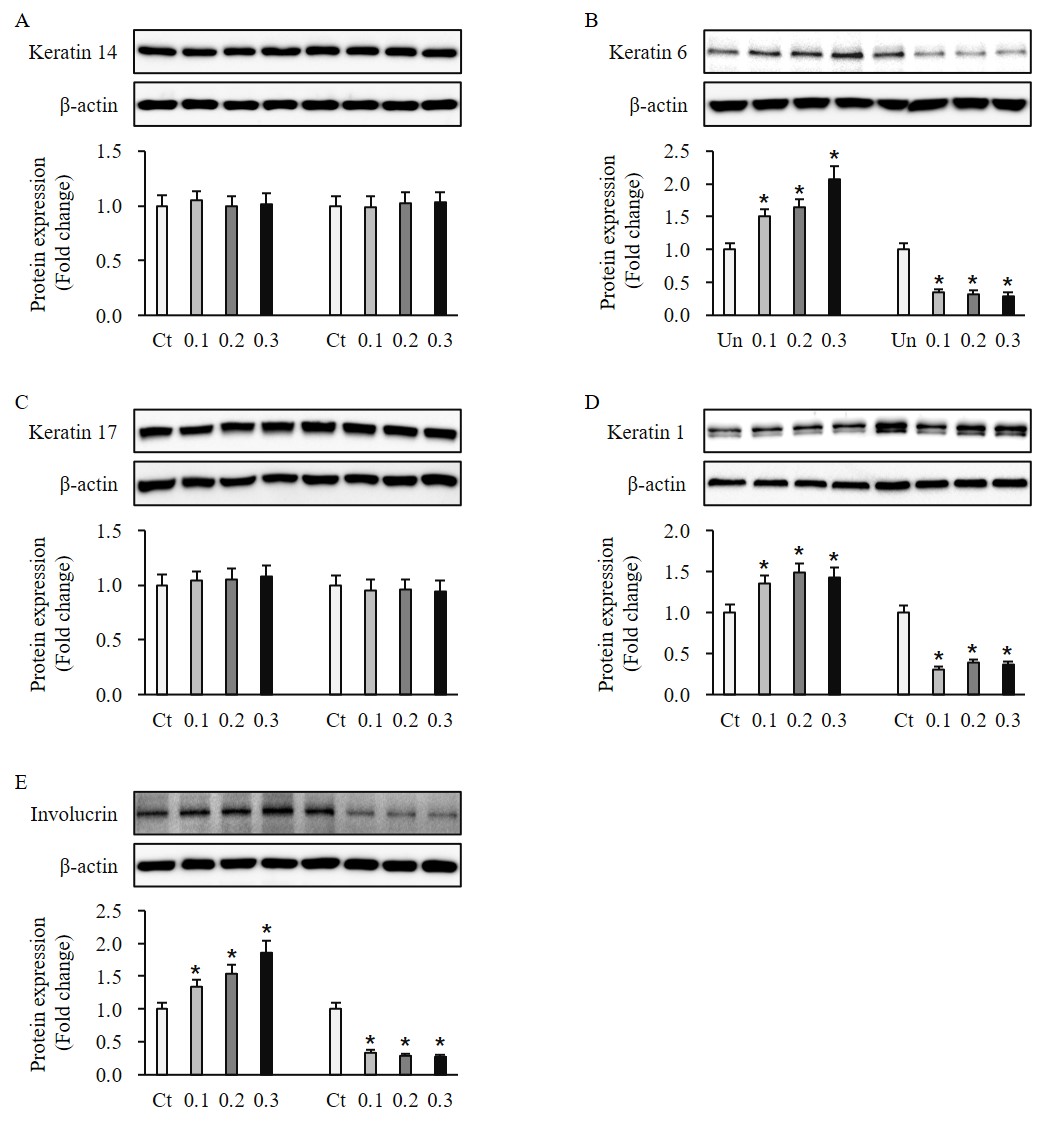


Figure S3. (A) Unchanged protein levels of keratin 14 in HNKs at 24h or 72h after ESWT under 0.1, 0.2, and 0.3 mJ/mm^2^ of energy flux density, comparted with those in untreated control. (B) Significantly increased and decreased protein levels of keratin 6 in HNKs at 24h or 72h after ESWT. (C) Unchanged protein levels of keratin 17 in HNKs at 24h or 72h after ESWT. (D) Significantly increased and decreased protein levels of keratin 1 in HNKs at 24h or 72h after ESWT. (E) Significantly increased and decreased protein levels of involucrin in HNKs at 24h or 72h after ESWT. In the fold change, untreated control cells marked as value 1; Ct, untreated control cells; HNK, human normal keratinocyte; **P* < 0.05 for ESWT-treated cells vs. the corresponding matched untreated control cells. Data represent means ± SD; n = 4.

Uncut western blot bands

Figure 1


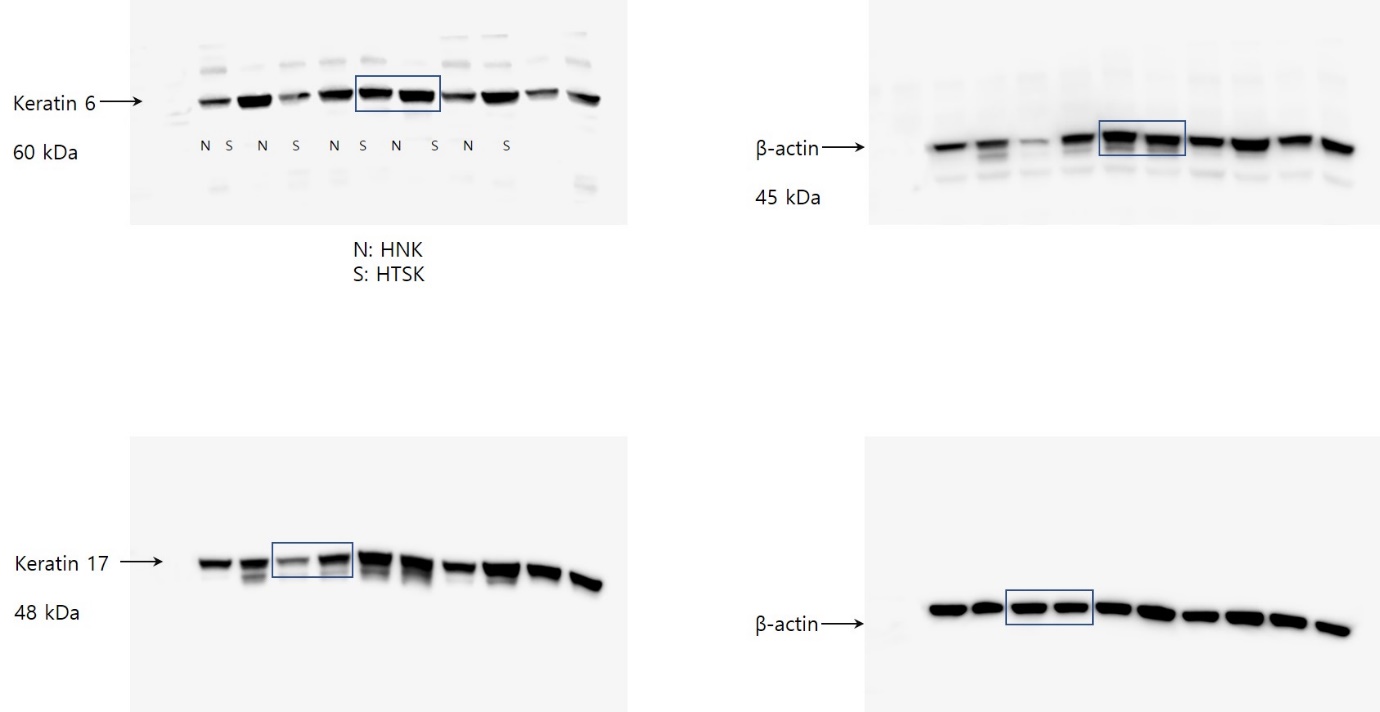


Figure 2


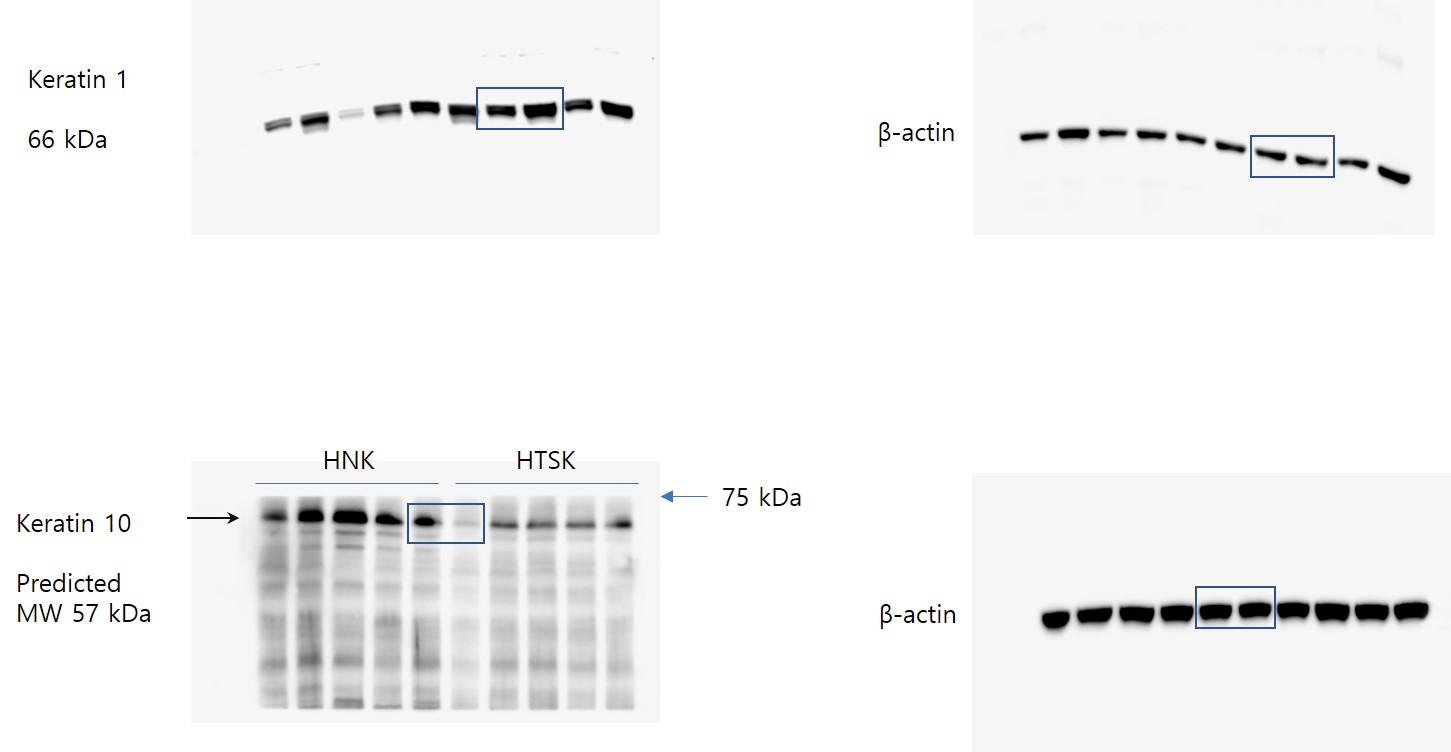


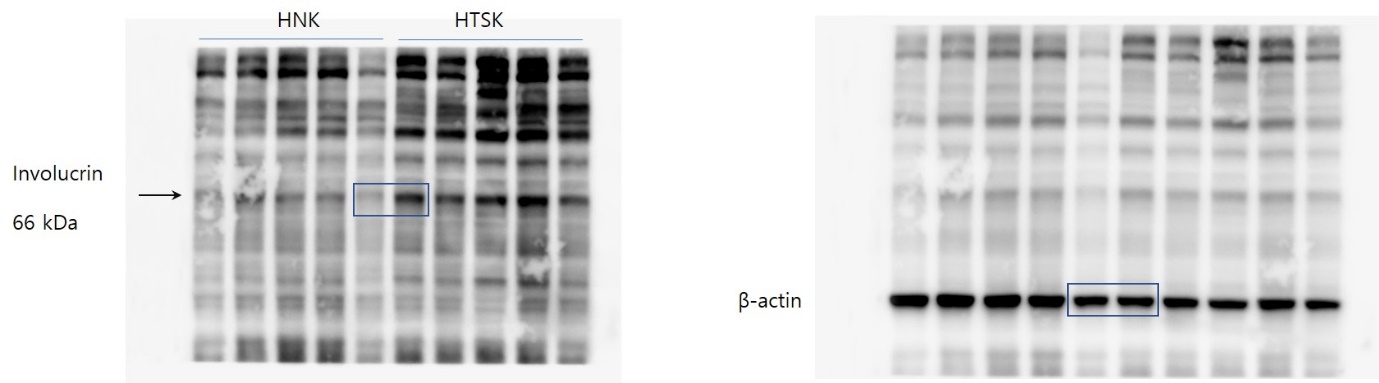


Figure 3


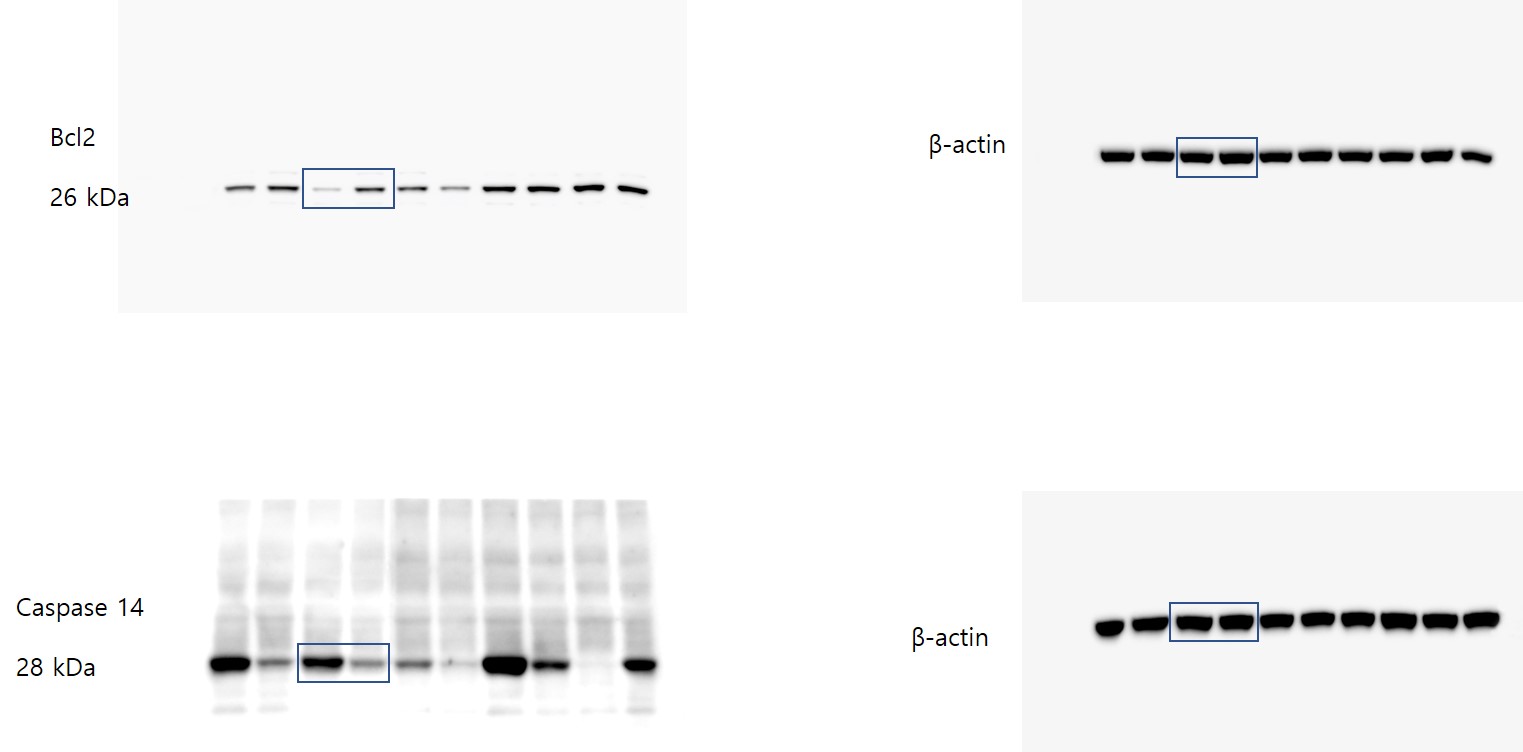


Figure 4


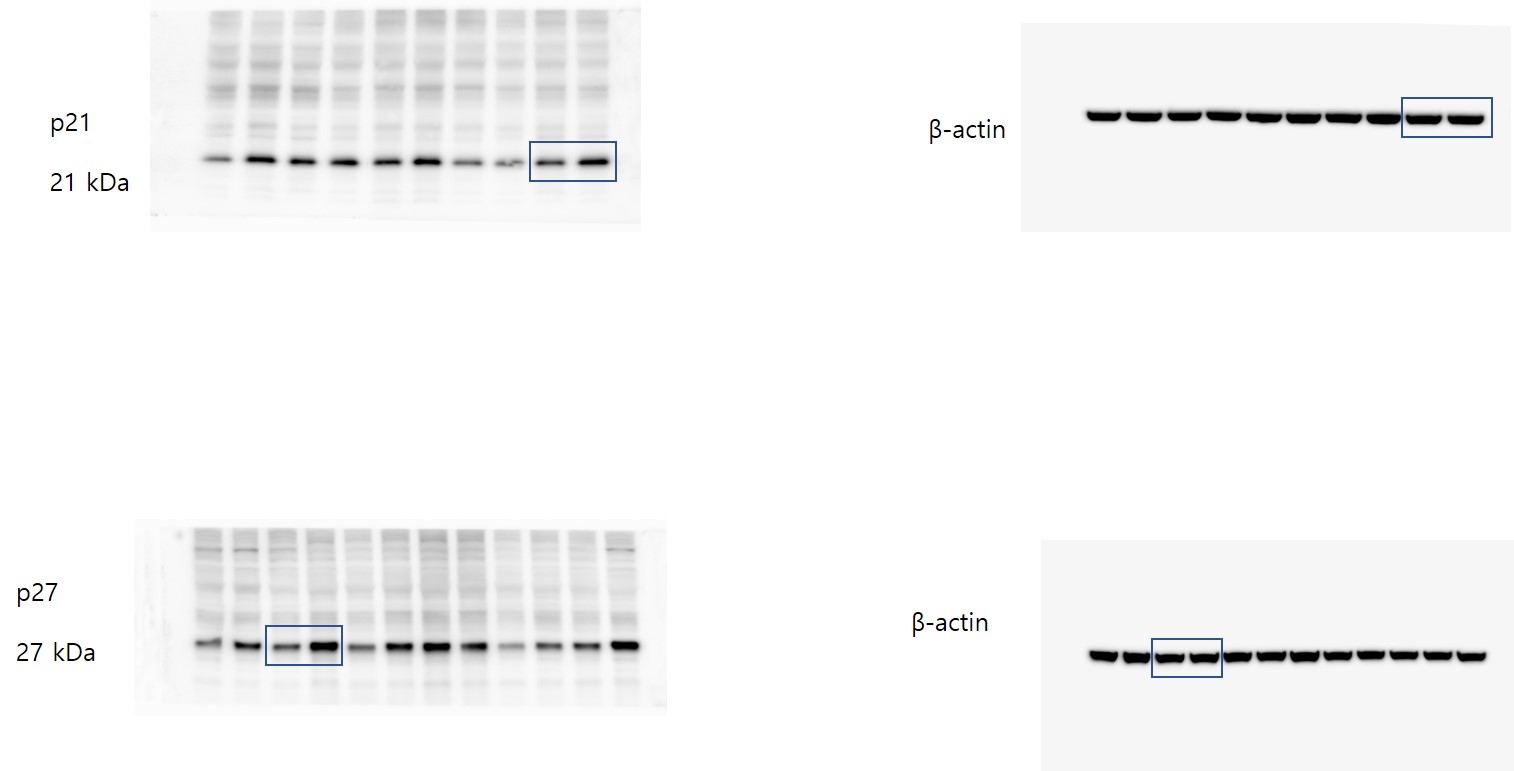


Figure 5


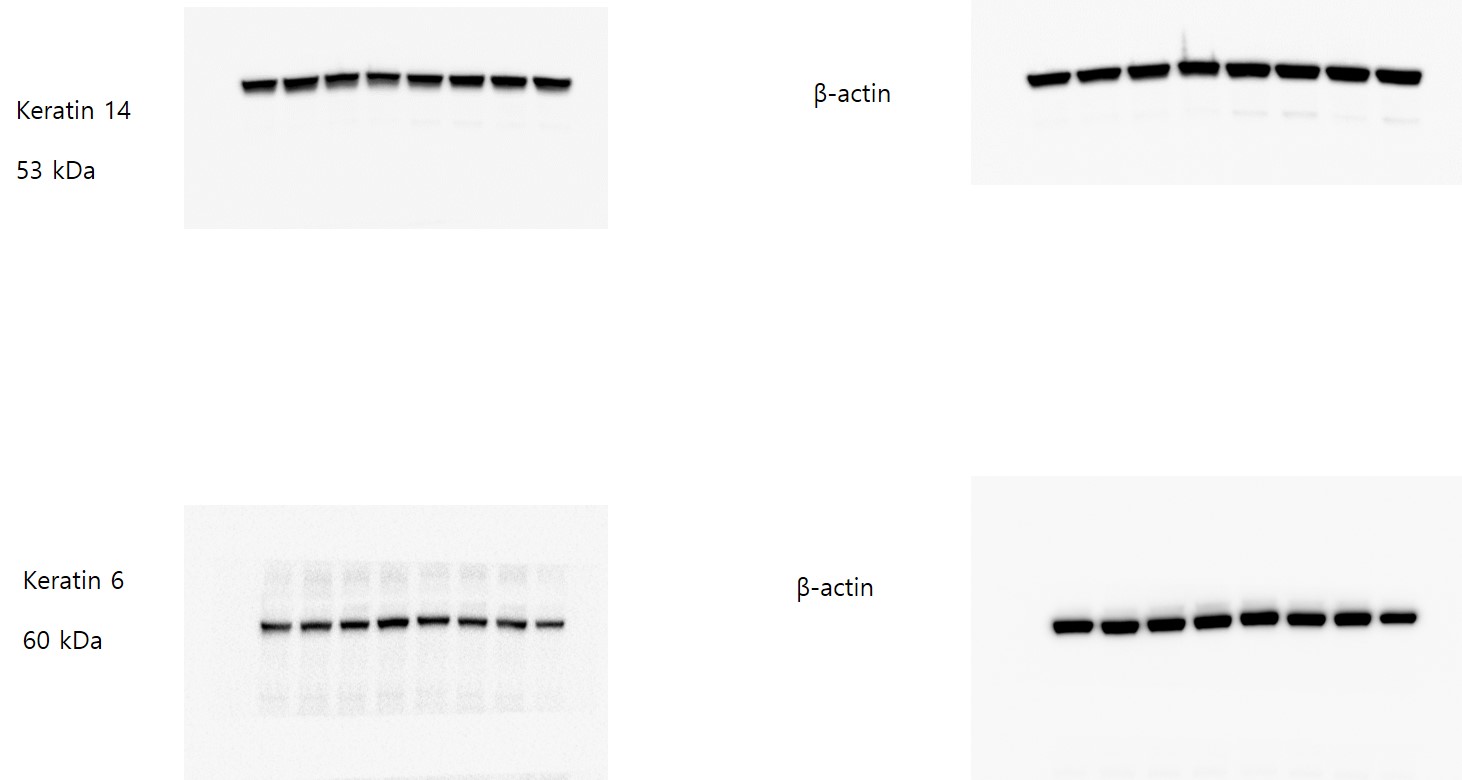


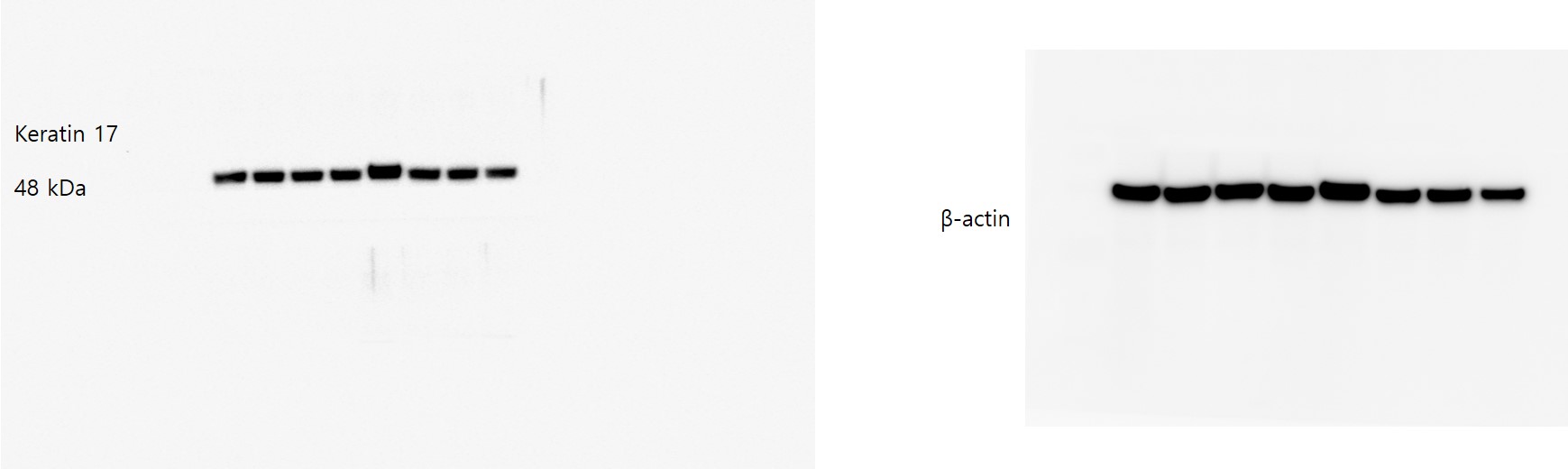


Figure 6


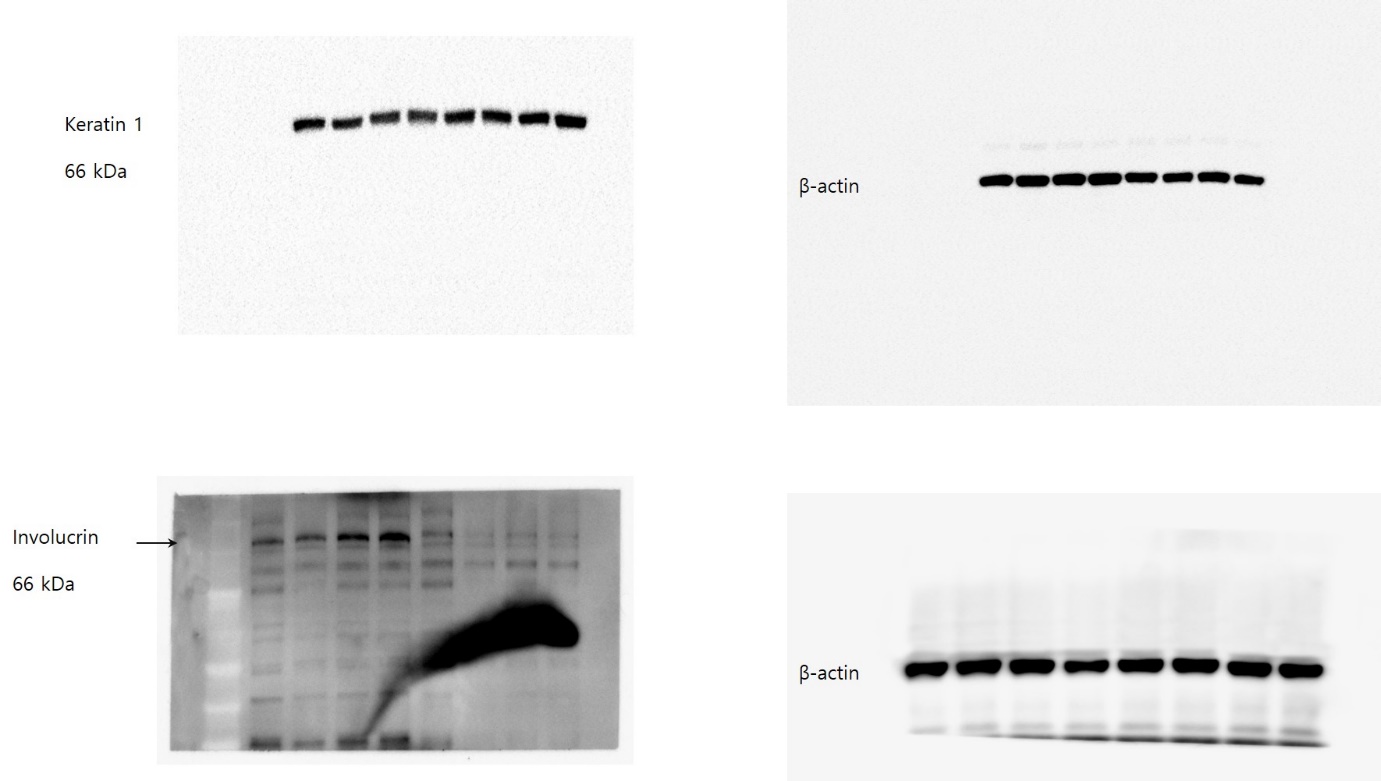


Figure 7


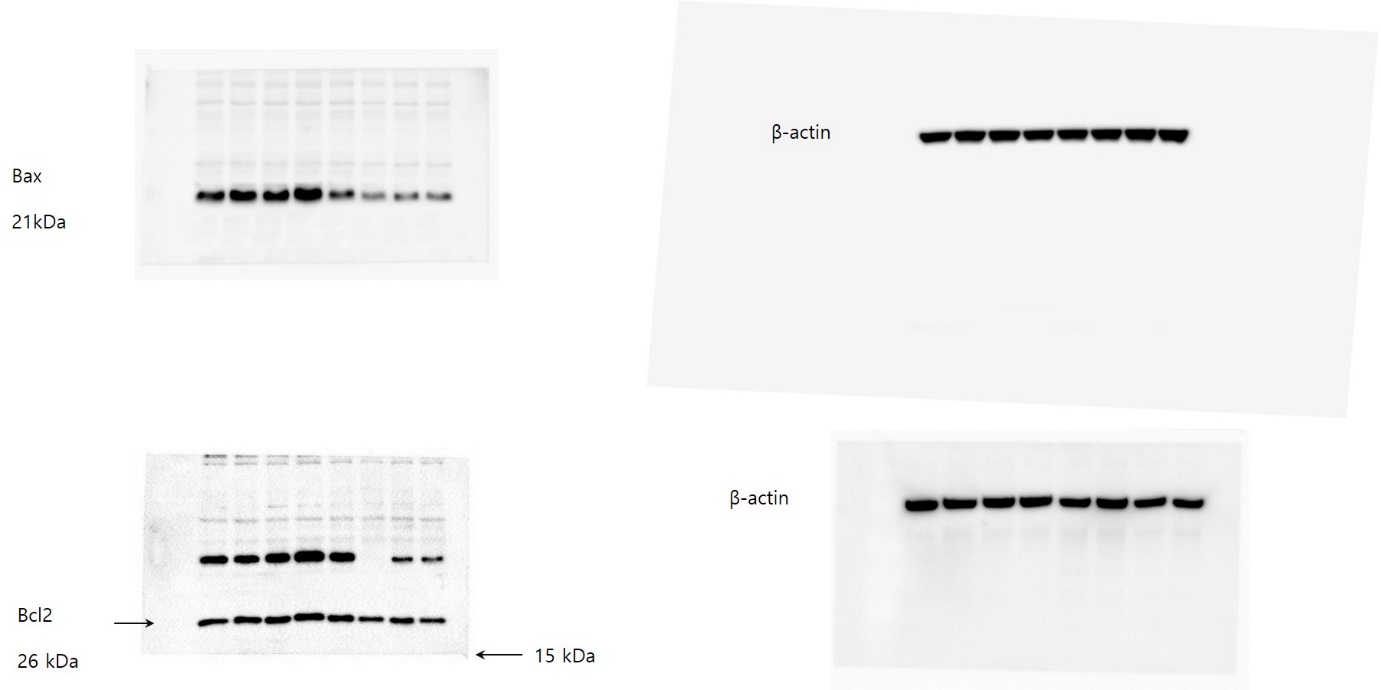


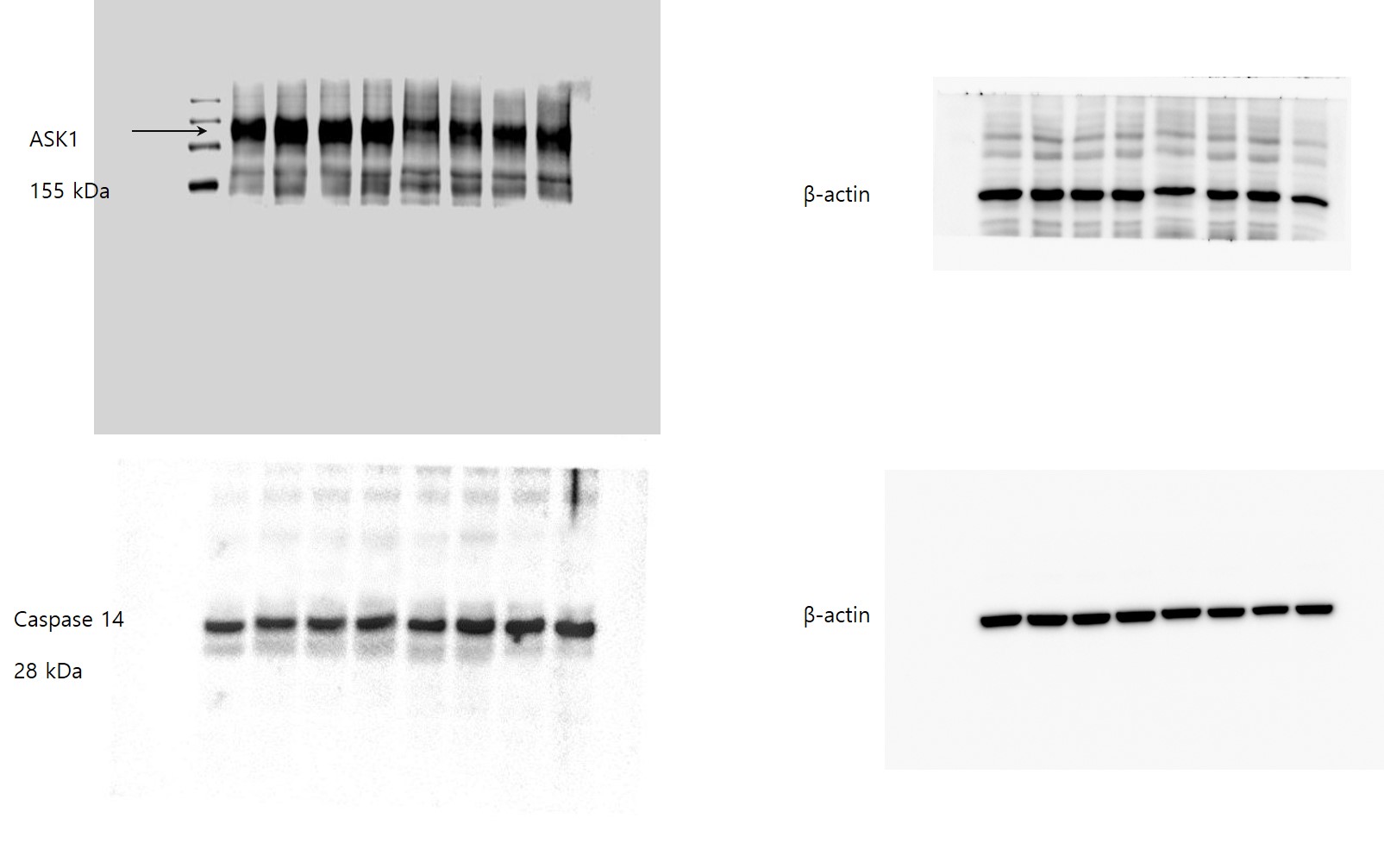


Figure 8


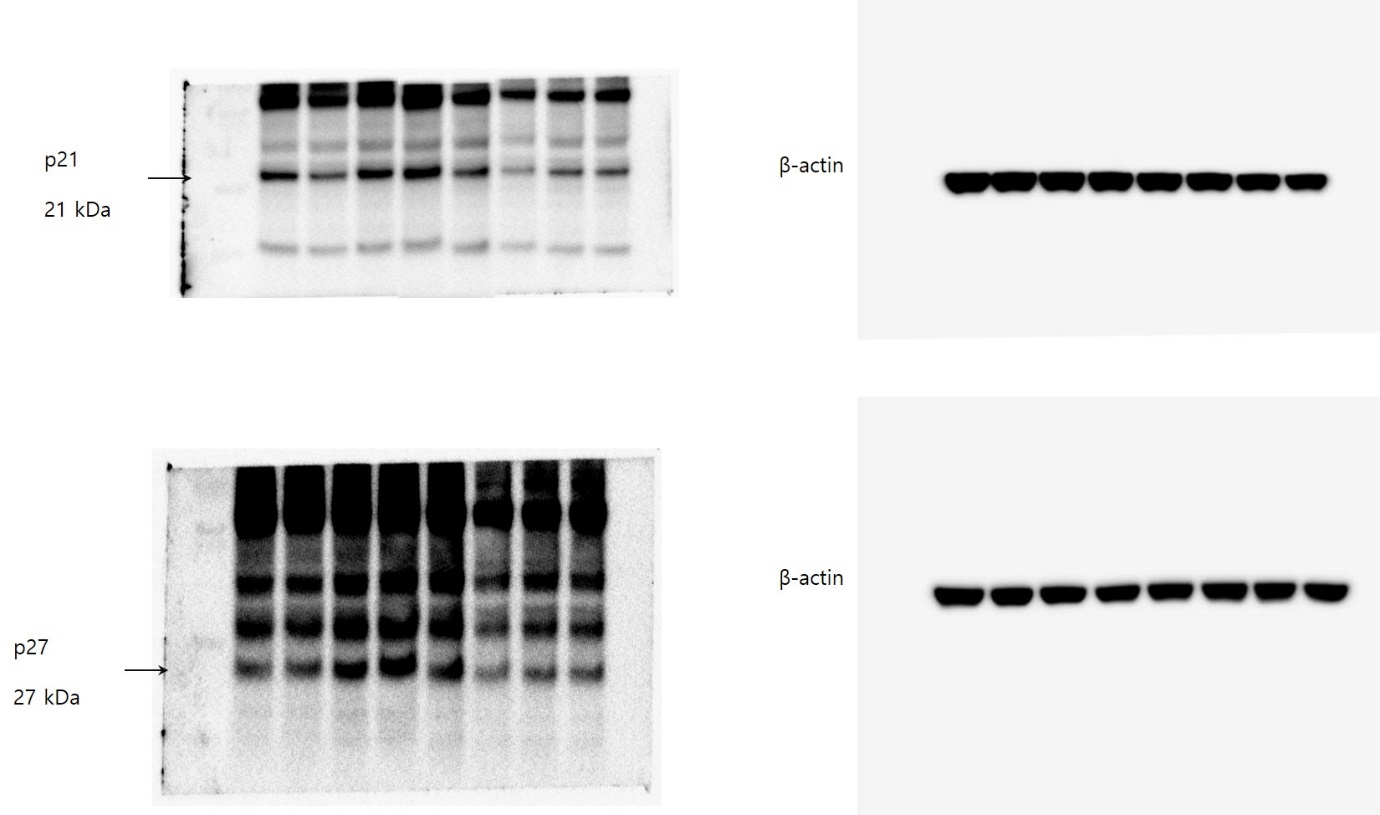


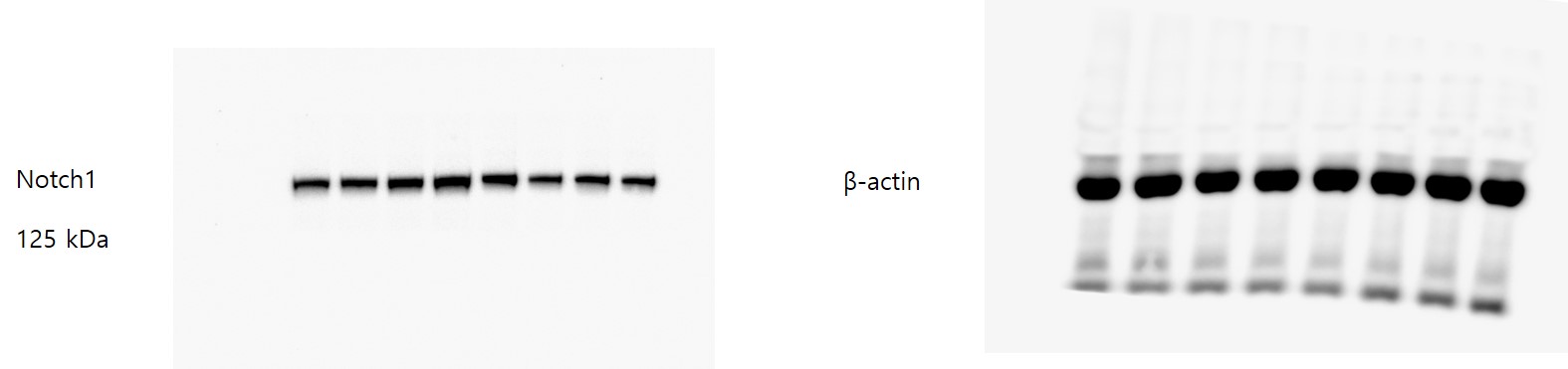


Figure S1


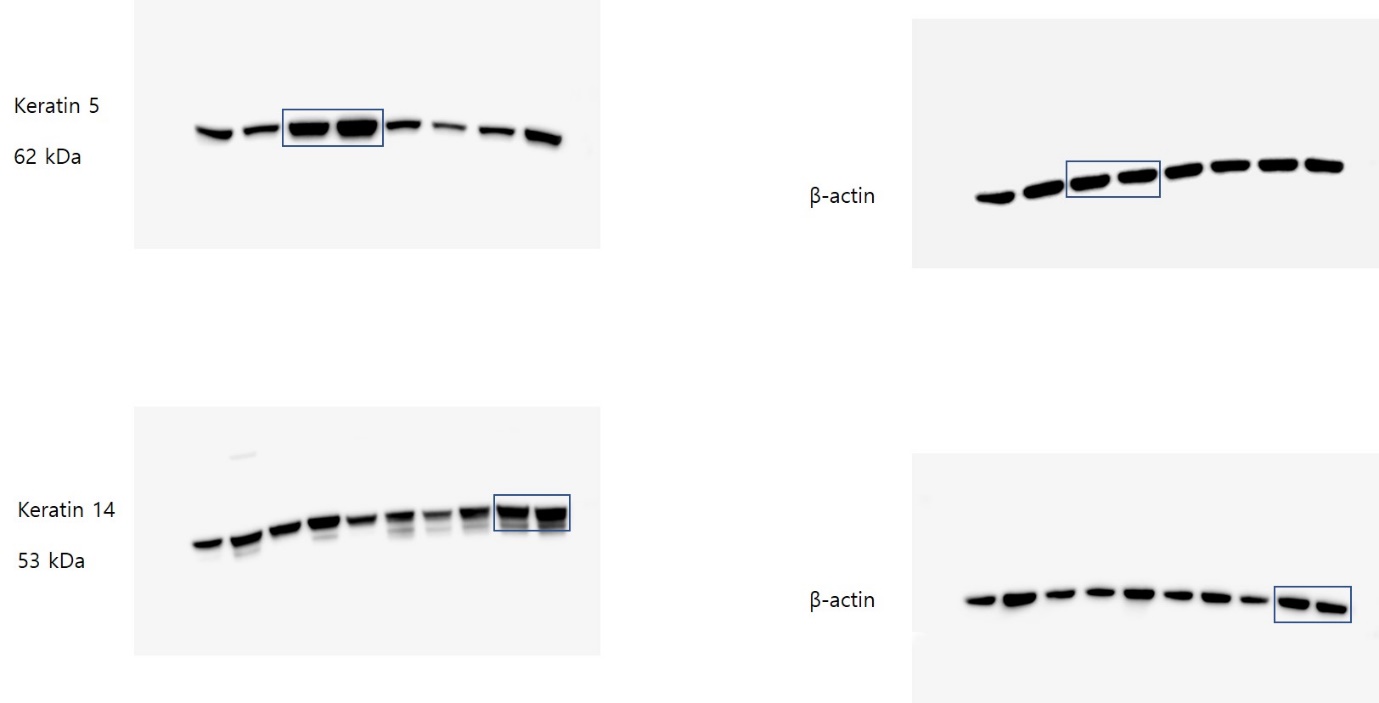


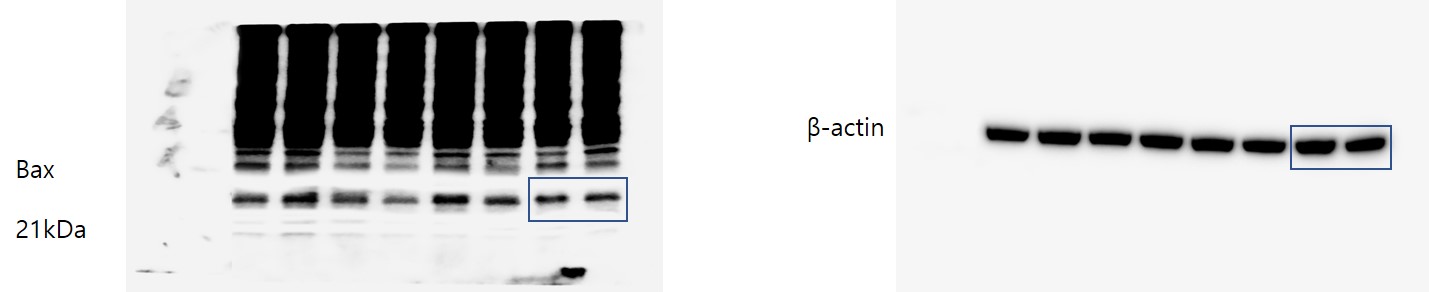


Figure S2


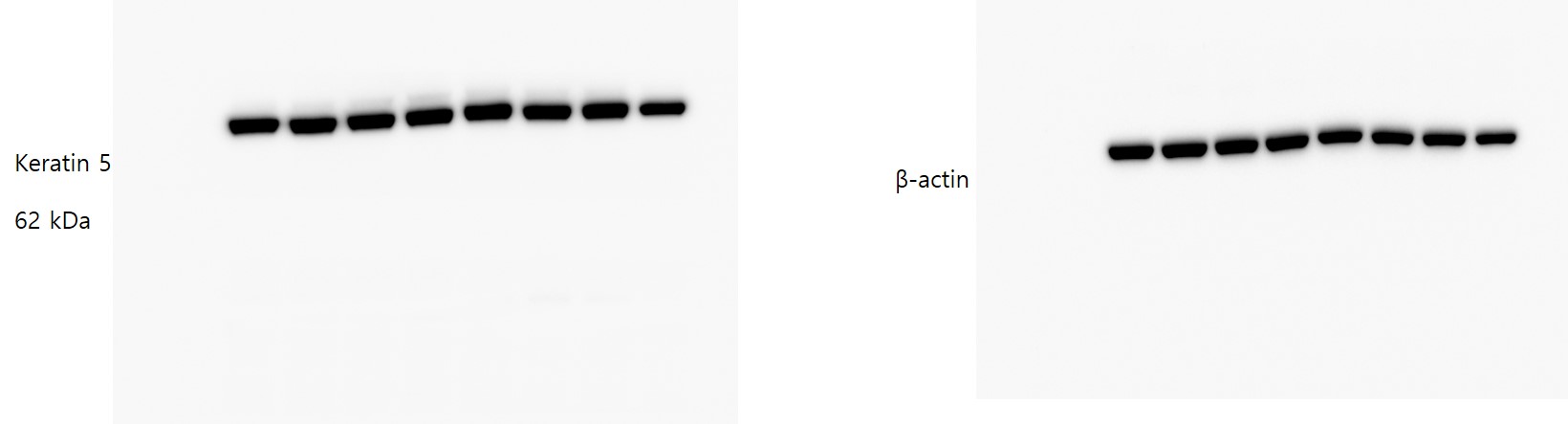


Figure S3


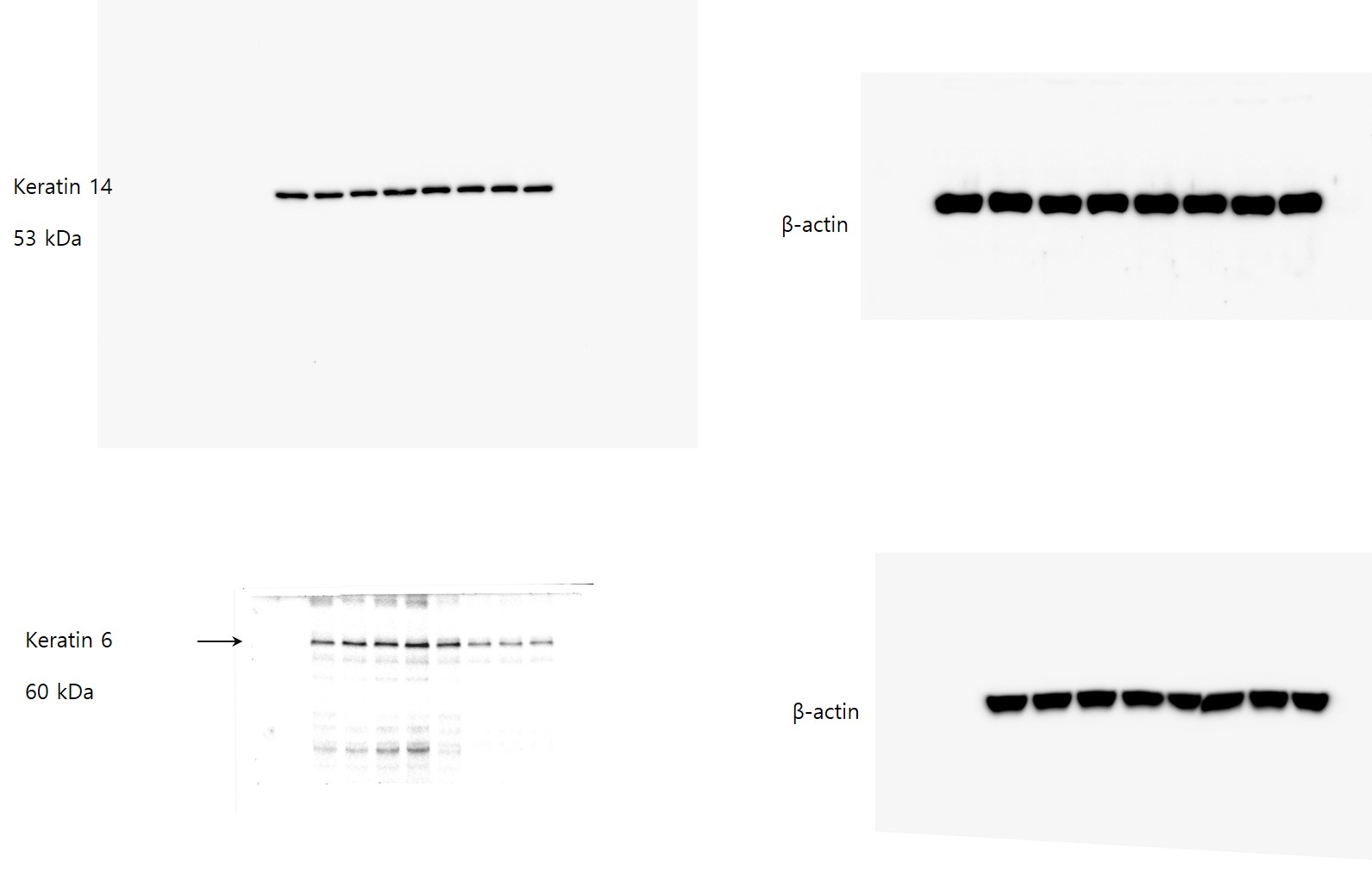


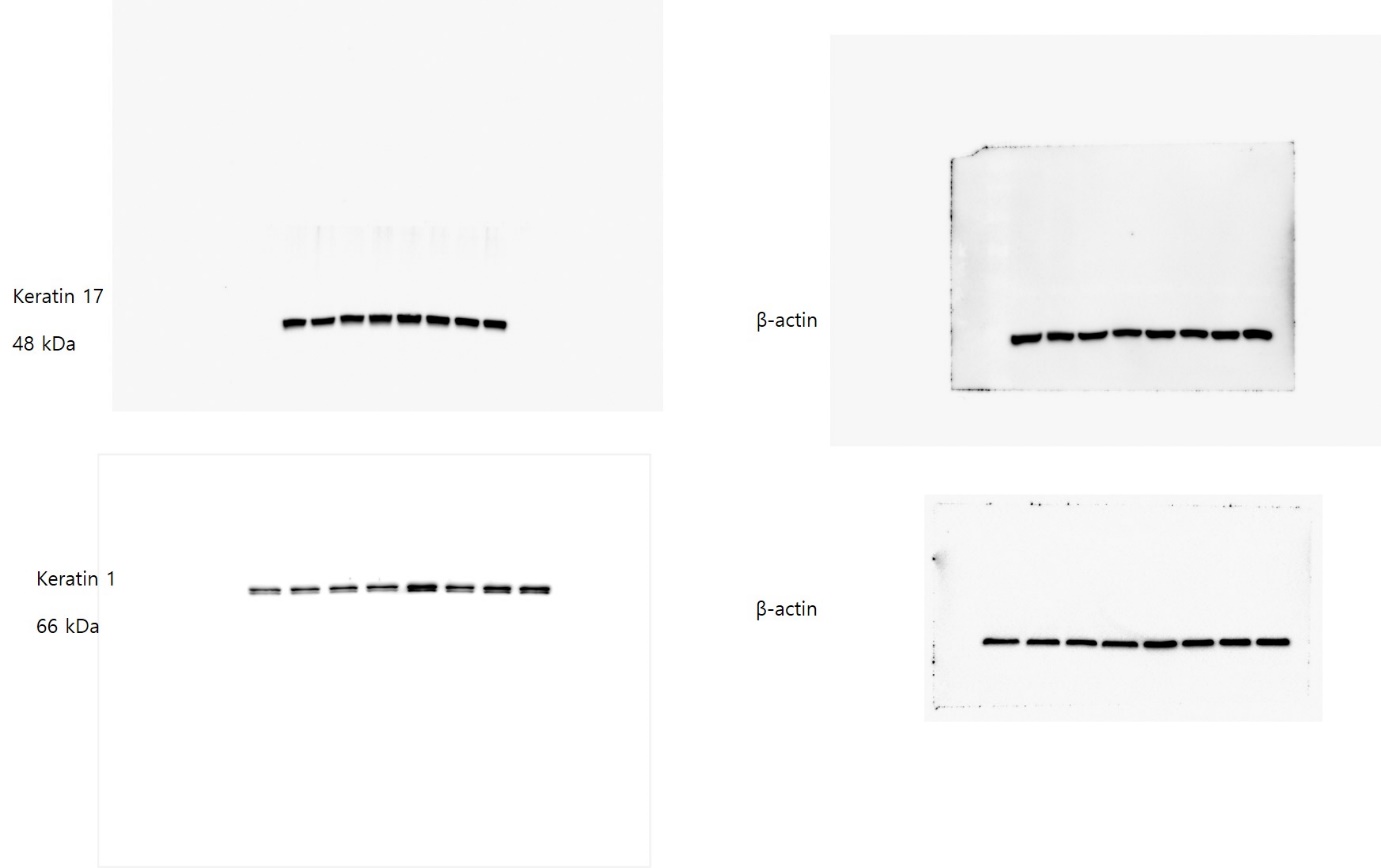


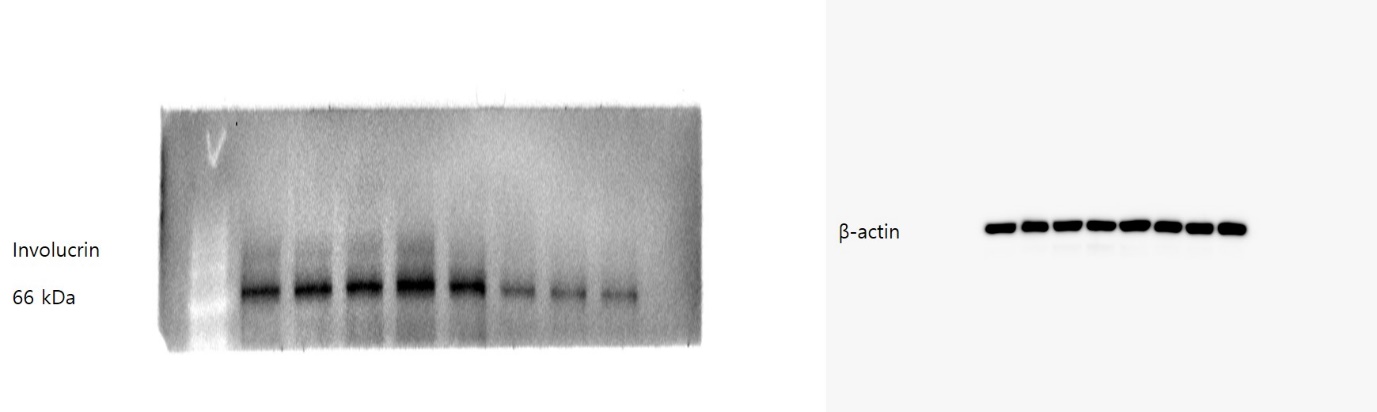

Supplement: Supplementary file 1 — Supplementary Figures. [file 41598_2021_96537_MOESM1_ESM.docx]
